# Diallel Analysis and Growth Parameters as Selection Tools for Drought Tolerance in Young *Theobroma cacao* Plants

**DOI:** 10.1371/journal.pone.0160647

**Published:** 2016-08-09

**Authors:** Emerson Alves dos Santos, Alex-Alan Furtado de Almeida, Dario Ahnert, Marcia Christina da Silva Branco, Raúl René Valle, Virupax C. Baligar

**Affiliations:** 1 Departamento de Ciências Biológicas, Universidade Estadual de Santa Cruz, Campus Soane Nazaré de Andrade, Rod. Jorge Amado, km 16, 45662-900, Ilhéus, BA, Brasil; 2 Centro de Pesquisas do Cacau, Comissão Executiva do Plano da Lavoura Cacaueira (CEPEC/CEPLAC), Rod. Jorge Amado, km 22, 45650-000, Ilhéus, BA, Brasil; 3 United States Department of Agriculture, Agricultural Research Service, Beltsville, MD, 20705-2350, United States of America; Agriculture and Agri-Food Canada, CANADA

## Abstract

This study aimed to estimate the combining ability, of *T*. *cacao* genotypes preselected for drought tolerance through diallel crosses. The experiment was conducted under greenhouse conditions at the Cacao Research Center (CEPEC), Ilhéus, Bahia, Brazil, in a completely randomized block design, in an experimental arrangement 21 x 2 [21 complete diallel crosses and two water regimes (control and stressed)]. In the control, soil moisture was kept close to field capacity, with predawn leaf water potential (Ψ_WL_) ranging from -0.1 to -0.5 MPa. In the drought regime, the soil moisture was reduced gradually by decreasing the amount of water application until Ψ_WL_ reached -2.0 to -2.5 MPa. Significant differences (p < 0.05) were observed for most morphological attributes analyzed regarding progenies, water regime and their interactions. The results of the joint diallel analysis revealed significant effects between general combining ability (GCA) x water regimes and between specific combining ability (SCA) x water regimes. The SCA 6 genetic material showed high general combining ability for growth variables regardless of the water regime. In general, the water deficit influenced the production of biomass in most of the evaluated *T*. *cacao* crosses, except for SCA-6 x IMC-67, Catongo x SCA, MOC-01 x Catongo, Catongo x IMC-67 and RB-40 x Catongo. Multivariate analysis showed that stem diameter (CD), total leaf area (TLA), leaf dry biomass (LDB), stem dry biomass (SDB), root dry biomass (RDB), total dry biomass (TDB), root length (RL), root volume (RV), root diameter (RD) <1 mm and 1 <(RD) <2 mm were the most important growth parameters in the separation of *T*. *cacao* genotypes in to tolerant and intolerant to soil water deficit.

## Introduction

Worldwide, drought is considered one of the most limiting factors for *T*. *cacao* production, being aggravated in recent years by global climate changes promoted by an increased greenhouse effect. Normally, plants under water deficit show low growth rates and photosynthesis, high root/shoot ratio, low capacity for nutrient absorption and lack of renewal of tissues, which provide plants with greater capacity to develop and overcome extreme environments [1, 2, 3]. In the specific case of *T*. *cacao*, growth and fruit production is regulated by the amount and distribution of rainfall [4]. *T*. *cacao* is considered to be slightly tolerant to water deficit [5, 6, 7]. Although it is typically grown in areas with high rainfall [8, 9], cacao growing regions are prone to periodic irregular rainfall, which may be aggravated by the predicted global climate changes [10].

Few studies have been conducted to identify *T*. *cacao* adaptation strategies to water deficit [11, 12, 13] and eventual use of water by the plant under such conditions [14]. It has been suggested that morphological changes are good indicators for early selection of cacao genotypes for tolerance to drought [15, 16, 17] due to the direct impact of drought on the plant growth and development patterns [18, 19]. The root system is one of the most sensitive organs of the plant to water limitation given the high capacity of the roots to recognize and respond to changes in the soil physicochemical parameters [20, 21]. Plants subjected to low soil water regimes can develop an extensive root system to capture the available soil water [22 23, 24, 25]. Furthermore, the limitation in soil water can also alter the partitioning of assimilates [15, 16], growth characteristics [23, 21], leaf production rate [22, 26] and leaf area [27] accelerating senescence and leaf drop [28]. However, limited information is available on the genetic control mechanisms, associated with all these characters in *T*. *cacao* subjected to drought.

The selection of germplasm with agronomic potential and knowledge about the inheritance of traits related to drought tolerance in *T*. *cacao* are fundamental in breeding programs, as they allow guiding crossings and development of segregating populations [29]. In this sense, diallel crossing is a genetic-statistical method that provides estimates of useful plant parameters for the selection of parents and to understand genetic effects involved in a given trait [30, 31].

For the selection of superior genotypes in segregating populations, the breeder needs to consider several characters altogether. For this, multivariate analyses techniques can be used. These procedures allow combining multiple information obtained in the experimental unit, facilitating the selection and discrimination of the most promising individuals. This strategy has been applied in *T*. *cacao*, especially in the study of genetic diversity [32, 33, 34, 35, 36, 37, 38] and drought tolerance [39]. However, in the present study parents with high combining ability for several morphological and growth characteristics, and related simultaneously to drought tolerance, will be used.

In this work, progenies of *T*. *cacao* from diallel crosses were subjected to drought in order to estimate, through univariate analyses, the effects of treatments and combining ability of genotypes and, through multivariate analyses identify useful growth and morphological characteristics for selection of drought tolerant parents.

## Materials and Methods

### Genetic material and experimental procedures

A total of seven *T*. *cacao* accessions, obtained from the Active Germplasm Bank of the Cacao Research Center (CEPEC) in Bahia, Brazil, were used in this study (Table 1). These accessions, which have been used as parents in breeding programs of *T*. *cacao* in Brazil, were crossed with each other by hand pollination in a diallel scheme to obtain progenies [40]. Recently, it was reported that these accessions have different levels of drought tolerance [39].

**Table 1:** *T*. *cacao* genotypes used in diallel crosses and its main characteristics.

Genotype	Origen	Leaf	Flower	Pod Index	Diseases	
			(N° ovules)	(Pods kg^-1^)	Witches’ Broom	Ceratocystis
SCA-6	Peru	C: 236 mm	42	47	R	S
		L: 70 mm				
CATONGO	Brazil	C: 293 mm	37	23	-	S
		L: 113 mm				
MOCORONGO	Brazil	-	-	28	-	S
PUCALA	Peru	-	-	23	-	-
IMC-67	Peru	C: 300 mm	48	22	S	S
		L: 91 mm				
TSH-1188	Trinidad	C: 236 mm	56	18	R	R
		L: 70 mm				
RB-40	Brazil	-	50	-	R	-

C—Length; L—width; R—Resistance; S—Susceptible. **Source:** International Cocoa Germplasm Database, 2015.

Sixty seeds of each of the 21 progenies of diallel crosses were picked randomly and planted in pots of 25 L, containing soil as substrate. The soil was analyzed for its physical and chemical characteristics and these results were used for fertilization purposes to provide adequate essential nutrients required to support good cacao seedling growth [41]. The experiment was conducted at the Cacao Research Center (CEPEC), Ilhéus, Bahia, Brazil (14° 47'S, 39° 16'W), under greenhouse condition during 2011-2012. Air temperature and relative humidity averages during the experimental period were 27±2°C and 80±3%, respectively. Based on a previous study, the maximum photosynthetic photon flux densities (PPFD) inside the greenhouse were between 1000 and 1200 μmol photons m^-2^ s^-1^.

Twelve months after sowing, the progenies were divided into two groups. Each group consisted of 126 plants to provide six plants /genotype. One group was subjected to water deficit by reducing the soil water content. Water, applied manually, was gradually suspended for 60 days until predawn leaf water potential (Ψ_WL_) reached -2.0 to -2.5 MPa. The other group was used as control, receiving daily irrigation to maintain soil moisture close to field capacity (33 kPa) and predawn Ψ_WL_ between -0.1 to -0.5 MPa.

The Ψ_WL_ measurements were taken on the second or third mature leaf from the top of the orthotropic axis between 02:00 and 04:00 h, using a PMS pressure chamber model 1000 (PMS Instrument Company, USA) according to methodology described by Scholander [42].

### Growth Parameters

For growth parameter determinations plants were sampled at two periods: (i) at the beginning of the dry cycle (12 months after sowing), when the Ψ_WL_ values for all crosses were between -0.1 to -0.5 MPa and soil moisture was close to field capacity and (ii) from 20 to 60 days after the drought began, when the Ψ_WL_ of the different crosses reached between -2.0 to -2.5 MPa. In both sampling dates, measurements were taken for: total (TLA) and individual (ILA) leaf areas, stem diameter (CD), plant height (H) and leaf number (LN) per plant. Leaf area was measured with a LI-COR 3100 area meter (Li-Cor, Inc. Lincoln, Nebraska, USA), CD and H with digital calipers and ruler, respectively.

Immediately after these evaluations, plants were removed from pots. The roots were washed 3x with demineralized water, placed in white plastic trays containing between 1.0–2.0 cm depth water lamina and photographed (Sony Lens 4x optical 12.1 Megapix). Soon after, each progeny was divided into root, stem and leaf, which were stored separately in paper bags and dried in a forced-air oven at 75°C to constant mass weight. These results were used to obtain: root (RDB), stem (SDB), leaf (LDB) and total dry biomass (TDB) of the plants.

Later, images of the plant root system were digitized in the Integrated System for Roots and Land Cover Analysis using the WinRhizo software, version 2013 (Regent Instrument, Quebec, Canada). After image processing, the following variables were obtained: root length (RL), root area (RA), mean root diameter (RD) and root volume (RV). Estimates of fine roots (RD < 1 mm), medium (1 < RD < 2 mm) and coarse (RD > 2.0 mm) classes were based on root studies of *T*. *cacao* [43]. Growth analysis procedures [44] were used to determine: specific leaf area (SLA), leaf mass ratio (LMR), leaf area ratio (LAR) and root/shoot (R/S) ratio.

### Statistical Analysis

We used a completely randomized design with 42 treatments (21 progenies x 2 water regimens (control—Ψ_WL_ between -0.1 to -0.5 MPa—and stressed—Ψ_WL_ between -2.0 and -2.5 MPa) and six replications. Analysis of variance (ANOVA) and estimates of genetic parameters were done considering the effects of progenies and water regime as fixed [45].

Progenies and parental means were grouped separately, according to Scott & Knott (P <0.05 and 0.01) and Tukey (P <0.05 and 0.01). Based on ANOVA results, sum of squares for treatments were broken into general combining ability (GCA) and specific combining ability (SCA), in accordance with method IV proposed by Griffing [46], in which only the F1 hybrids are included, with a total of p(p-1)/2 combinations. The following statistical model Ῡ_ij_ = μ + g_i_ + g_j_ + s_ij_ + ε_ij_, was used, where: Y_ij_ = progenies mean of crosses between the i-th and j-th parent in r replications, μ = general mean, g_i_ and g_j_ = effect of the general combining ability of the i-th and j-th parental, s_ij_ = effect of the specific combining ability for the progenies between parents of order i and j, respectively, εij = mean experimental error.

### Multivariate analysis

For discrimination of genetic materials in the different water regimes, the following multivariate analyses were used: factorial and biplot analyses based on principal component (PC). Initially 20 growth variables (H, CD, TLA, LN, ILA, RDB, LDB, SDB, TDB, LAR, SLA, LMR, R/S, RL, RA, RD, RV, RD < 1 mm, 1 < RD <2 mm and RD > 2 mm) were standardized due to differences in units. Standardization was based on the equation Z_ij_ = (X_ij_-μ_j_)/S_j_, where X_ij_ is the value of i-th observation of variable X_j_, μ_j_ and S_j_ are the mean and standard deviation of X_j_, respectively.

All standardized variables were subjected to factorial analysis, using the computing environment R, version 3.03 for Windows [47]. The results showed that only 13 variables were considered relevant for the formation of the first three factors (total variation 70%). These variables were then submitted to collinearity analysis based on tolerance and the variance inflation factor, considering, as the threshold for inclusion, values greater than 0.1 and less than 10, respectively [48].

The variables considered non-collinear (CD, TLA, LDB, SDB, RDB, TDB, RL, RV, RD <1 mm and 1 < RD <2 mm) were used for biplot analysis. For this analysis, a double entry table progenies x non-collinear variables in different water regimes was subjected to an analysis of decomposition of singular value, and the scores of the first two principal components used as coordinates to plot, according to the model described by Yan and Rajcan [49]: (*A*_*ij*_*− Ᾱ*_*ij*_)/*s*_*ij*_ = *λ*_*1*_*ξ*_*i1*_*τ*_*j1*_ + *λ*_*2*_*ξ*_*i2*_*τ*_*j2*_ + *ε*_*ij*_, where, *A*_*ij*_ = value observed in the ith progeny and i-th variable; *Ᾱ*_*ij*_ = mean of the ith progeny in variable j; λ_1_ and λ_2_ = singular values for the first (PC1) and second principal components (PC2), respectively; *ξ*_*1*_ and *ξ*_*2*_ = scores of PC1 and PC2 for the progeny; *τ*_*1*_ and *τ*_*2*_ = scores of PC1 and PC2 for variable j; *ε*_*ij*_ = model residue associated with progeny and variable and *s*_*ij*_ = standard deviation of the estimate.

In constructing the biplot it was decided to group the progenies by category in agreement to the water regime. In the water stress condition, the progenies were grouped in two ways: (1) progenies tolerant to drought—showed mean values greater than the overall mean for more than 60% of the analyzed variables; and (2) progenies non-tolerant to drought—showed mean values below the overall mean for more than 60% of the analyzed variables. In the control condition (no stress), it was decided to cluster the progenies also in two groups: (1) high vegetative vigor—those that showed mean values above the overall mean for more than 60% of the variables; and (2) low vegetative vigor—the ones with mean values below the overall mean for more than 60% of the analyzed variables.

## Results

The individual analysis of variance revealed significant differences (p ≤ 0.05) among the means of the progenies for the different shoot and root morphological attributes evaluated, showing the existence of genetic variation among the parents used in the diallel crosses (Table 2). Such responses were expected since differences exists among the parental lines used and they are from different geographical origins. Regarding the effect of soil water treatments, there were no significant differences observed for ILA, SDB, RDB, RL and RD < 1 mm, suggesting that the performance of progenies for different soil moisture levels varied depending on environment in which they are assessed.

**Table 2:** Analysis of variance of 21 progenies of a full diallel (PROG) for shoot and root morphological attributes used in the analysis of cacao genotypes contrasting for drought tolerance submitted for 60 days to two water conditions (SI): Control [Ψ_WL_ between -0.1 and -0.5 MPa] and stressed (Ψ_WL_ between -2, 0–2.5 MPa). Ilhéus, Bahia, Brazil.

Source of Variation	DoF	H	CD	TLA	LN	ILA	LDB	SDB	TDB	LAR	SLA	LMR	RDB	R/S	RL	RA	RD	RV	RD < 1	1< RD <2	RD >2
		(m)	(cm)	(m^2^)	(unit.)	(m^2^.plant^-1^)	(g.plant^-1^)	(g.plant^-1^)	(g.plant^-1^)	(m^2^. g^-1^)	(m^2^. g^-1^)	(g/g)	(g.plant^-1^)		(cm)	(cm^2^)	(mm)	(cm^3^)	(mm)	(mm)	(mm)
PROG	20	0.4***	36.4***	25922308.0*	1241.0***	7094.0 ***	1124.0***	2034.0***	7788.0***	610.0***	2439.0***	0.0***	310.0***	0.0**	2740921.0***	662209.0***	0.8***	41602.0***	316373.0**	229150.0***	505578.0***
SI	1	0.6**	135.0***	73566219.0***	1472.0***	145.0^ns^	10764.0**	23.4^ns^	13792.0**	15176.0***	13793.0***	0.1***	76.4 ^ns^	0.1***	1296967.0^ns^	5775153.0***	37.1***	1050501.0***	4960373.0***	108150.0^ns^	12021085.0***
PROG x SI	20	0.1**	19.3*	25771938.0**	152.0^ns^	3476.0**	417.0**	822.0**	2550.0*	435.0**	1553.0***	0.0^ns^	164.0**	0.0**	2026416.0**	234308.0**	0.6**	25034.0 **	325898.0**	310693.0***	315837.0*
Error	210	0.1	11	10427373	115	1621	181	353	1522	220	569	0	72.5	0.3	888901	116303	0.3	10905	142165	95446	180697

H, high; CD stem diameter; TLA, total leaf area; NL, leaves number; ILA, individual leaf area; LDB, leaf dry biomass; SDB, stem dry biomass; TDB, total dry biomass; LAR, leaf area ratio; SLA, specific leaf area; LMR, leaf mass ratioRDB, root dry biomass; R/S root/shoot ratio; RL, root length; RA, root area; RD, mean root diameter; RV, root volume; RD, fine root diameter (RD < 1 mm), medium (1 < RD < 2 mm) and coarse (RD > 2 mm).

*** Significance by the F test at p < 0.001

** p < 0.01

* p < 0.05.

In the joint analysis of variance, the interaction progeny x water regime was significant (p <0.05) in almost all the analyzed plant characteristics, except for LN and LMR, indicating that there were differential progeny responses with respect to the tested soil water regimes. These results were also expected given the wide difference in water regimens and genetic variation of the parents. The coefficients of variation (CVs) ranged from 13.7% to 38.8% for LMR and RV, respectively (Table 3).

**Table 3:** Estimates of genetic parameters for shoot and root morphological attributes used in the analysis of cacao genotypes contrasting for drought tolerance submitted for 60 days to two water conditions (SI): Control (ΨWL between -0.1 and -0.5 MPa) and stressed (ΨWL between -2, 0–2.5 MPa). Ilhéus, Bahia, Brazil.

Parameters										Morphological Attributes										
	H	CD	TLA	LN	ILA	LDB	SDB	TDB	LAR	SLA	LMR	RDB	R/S	RL	RA	RD	RV	RD<1	1<RD<2	RD>2
CV_e_ (%)	14.1	16.1	20.6	33.4	20.3	17.0	27.2	23.4	22.0	16.5	13.7	25.4	17.8	26.5	28.9	25.9	38.8	29.0	28.4	36.0
CV_g_ (%)	7.1	7.1	10.4	19.1	10.8	14.2	17.5	13.7	8.5	7.8	5.4	13.3	8.6	12.1	24.8	7.4	29.0	7.5	9.0	17.0
h_a_^2 (%)^	60.3	69.9	46.4	79.6	77.1	82.8	78.2	80.5	64.0	72.9	65.5	76.6	43.6	65.0	76.5	39.1	69.4	43.0	36.9	57.8
CV_g_/CV_e_	0.5	0.4	0.3	0.6	0.5	0.6	0.6	0.6	0.4	0.5	0.4	0.5	0.3	0.4	0.5	0.2	0.4	0.3	0.2	0.3

(CV_e_), experimental variation coefficient; (CV_g_), genetic variation coefficient and (h_a_^2^) heritability H, high; CD stem diameter; TLA, total leaf area; NL, leaves number; ILA, individual leaf area; LDB, leaf dry biomass; SDB, stem dry biomass; TDB, total dry biomass; LAR, leaf area ratio; SLA, specific leaf area; LMR, leaf mass ratio; RDB, root dry biomass; R/S root/shoot ratio; RL, root length; RA, root area; RD, mean root diameter; RV, root volume; RD, fine root diameter (RD < 1 mm), medium (1 < RD < 2 mm) and coarse (RD > 2 mm)

The high heritability (h^2^) values observed for the variables LDB (83%), LN (80%) TDB (80%), SDB (78%), RDB (77%), ILA (77%), RA (76%) SLA (73%), CD (70%), RV (69%) and RL (65%) reflect the low environmental influence on these attributes, which may allow greater genetic gain in the selection process (Table 3). The CVg/CVe values shown by LDB (0.6), TDB (0.6), LN (0.6), SDB (0.6), ILA (0.5), RDB (0.5) and R/A (0.5) indicate sufficient genetic variability that justifies continuity of the breeding program.

In general, soil water deficit significantly (p<0.05) influenced biomass production, reducing dry weight in all plant parts for most of the evaluated cacao genotypes (Table 4). Significant reductions (P<0.05) in root (RDB), stem (SDB), leaf (LDB) and total (TDB) dry biomass were found in the genetic material in relation to their controls, except for SCA-6 x IMC-67, RB-40 x IMC-67, Catongo x SCA-6, MOC-01 x Catongo, Catongo x IMC-67 and RB-40 x MOC-01. In contrast, PUCALA x SCA-6, PUCALA x Catongo, MOC-01 x IMC-67 and PUCALA x MOC-01 were the progenies with more significant reductions both shoot and root system under soil water deficit.

**Table 4:** Mean values for growth and morphological attributes associated with the shoot and root of F1s in the control condition (Ψ_WL_ between -0.1 and -0.5 MPa) and stress condition (Ψ_WL_ between -2.0 and -2.5 Mpa). Ilhéus, Bahia, Brazil.

Progenies	Treatment	H	CD	TLA	LN	ILA	LDB	SDB	TDB	LAR	SLA	LMR	RDB	R/S	RL	RA	RD	RV	RD<1	1<RD< 2	RD>2
		m	cm	m^2^	unid	m^2^.plant^-1^	g.plant^-1^	g.plant^-1^	g.plant^-1^	(m^2^. g^-1^)	(m^2^. g^-1^)	(g/g)	g.plant^-1^		cm	cm^2^	mm	cm^3^	mm	mm	mm
CATONGO x SCA 06	Control	2.0 ± 0.0	25.1 ± 2	1.2 ± 0.1	62.8 ± 3*	0.0 ± 0.0	78.0 ± 8.1	71.8 ± 5	194.1 ± 19	0.0 ± 0.0	0.0 ± 0.0	0.3 ± 0.0	30.2 ± 1.7	0.2 ± 0.0	353 ± 30	930 ± 45	2.5 ± 0.2	197 ± 40	1564 ± 115	941 ± 77	1051 ± 81
	Drought	2.1 ± 0.1	21.3 ± 1	1.0 ± 0.1	49.3 ± 3	0.0 ± 0.0	76.86 ± 5.3	96.7 ± 9*	169.6 ± 16	0.0 ± 0.0	0.0 ± 0.0	0.3 ± 0.0	36.9 ± 4.4	0.3 ± 0.0*	333 ± 37	706 ± 88	1.9 ± 0.2	127 ± 45	1732 ± 114	877 ± 80	795 ± 44
PUCALA x SCA 06	Control	1.9 ± 0.1*	21.7 ± 1*	1.1 ± 0.1*	50.1 ± 1***	0.0 ± 0.0	63.5 ± 3.6**	66.3 ± 3*	170.8 ± 7*	0.0 ± 0.0*	0.0 ± 0.0	0.4 ± 0.0*	35.5 ± 4.1*	0.3 ± 0.0	238 ± 32	845 ± 26	2.5 ± 0.3*	256.8 ± 34*	1361 ± 123	845 ± 95	1092 ± 90*
	Drought	1.7 ± 0.0	18.8 ± 1	0.7 ± 0.1	30.6 ± 3	0.0 ± 0.0	37.5 ± 4.4	51.7 ± 5	149.3 ± 7	0.0 ± 0.0	0.0 ± 0.0	0.3 ± 0.0	24.9 ± 1.9	0.3 ± 0.0	326 ± 24*	469± 54	1.5 ± 0.1	51 ± 2.7	1849 ± 146*	868 ± 65	458 ± 57
TSH 1188 x SCA 06	Control	2.1 ± 0.0	20.0 ± 1	1.7 ± 0.3**	82 ± 3.2**	0.0 ± 0.0	65.9 ± 2.3**	66.1 ± 6	163.0 ± 9	0.0 ± 0.0	0.0 ± 0.0	0.4 ± 0.0**	30.4 ± 1.9	0.2 ± 0.0	437 ± 17	1261 ± 86	2.7± 0.3	355.0 ± 33	1723 ± 39	1233 ± 120	1650 ± 91*
	Drought	2.0 ± 0.0	21.0 ± 2	0.5 ± 0.0	52.2 ± 6	0.0 ± 0.0	37.2 ± 0.9	64.6 ± 5	154.4 ± 9	0.0 ± 0.0	0.0 ± 0.0	0.3 ± 0.0	31.3 ± 4.6	0.3 ± 0.0	374 ± 26	841 ± 39	2.3 ± 0.1	153 ± 16	1772 ± 147	1057 ± 76	895 ± 31
RB 40 x SCA 06	Control	2.0 ± 0.1	25.2 ± 2	1.3 ± 0.1	64 ± 7	0.0 ± 0.0	78.3 ± 7.5	79.8 ± 8	214.8 ± 29	0.0 ± 0.0	0.0 ± 0.0	0.4 ± 0.0	39.8 ± 3.9	0.2 ± 0.0	387 ± 45	950 ± 26*	2.4 ± 0.4*	205.6 ± 34	1931 ± 123	1100 ± 105*	1650 ± 91*
	Drought	2.0 ± 0.1	21.6 ± 1	0.9 ± 0.1	55.3 ± 3	0.0 ± 0.0	63.9 ± 3.3	90.0 ± 5	189.1 ± 7	0.0 ± 0.0	0.0 ± 0.0	0.4 ± 0.0	33.4 ± 3.3	0.2 ± 0.0	294 ± 26	457 ± 18	1.5 ± 0.1	66 ± 12	1805 ± 112	770 ± 26	895 ± 31
MOC 01 x SCA 06	Control	1.9 ± 0.0	21.3 ± 1	1.3 ± 0.1	57.6 ± 4	0.0 ± 0.0	70.0 ± 6.9	79.5 ± 5**	177.1 ± 6	0.0 ± 0.0	0.0 ± 0.0	0.4 ± 0.0	34.4 ± 4.5	0.2 ± 0.0	383 ± 32	1083 ± 84	2.8 ± 0.2**	323.7 ± 34*	1412 ± 97*	901 ± 85	1446 ± 176*
	Drought	2.1 ± 0.0	17.1 ± 3	1.0 ± 0.1	59.5 ± 4	0.0 ± 0.0	60.7 ± 3.9	51.6 ± 5	154.1 ± 7	0.0 ± 0.0	0.0 ± 0.0	0.4 ± 0.0	27.6 ± 1.5	0.2 ± 0.0	367 ± 23	640 ± 28	1.7 ± 0.1	90.4 ± 18	2009 ± 131	969 ± 65	689 ± 24
SCA 06 x IMC 67	Control	2.0 ± 0.1	23.7 ±2	1.3 ± 0.1	66.3 ± 3	0.0 ± 0.0	79.8 ± 6.1	71.8 ± 5	224.8 ± 21	0.0 ± 0.0	0.0 ± 0.0	0.4 ± 0.0	42.5 ± 4.8	0.2 ± 0.01	405 ± 29*	1620 ± 49*	3.6 ± 0.2**	458 ± 50 *	1489 ± 113	1000 ± 76	1558 ± 76
	Drought	2.1 ± 0.1	24.2 ± 1	1.1 ± 0.1	53.7 ± 5	0.0 ± 0.0	71.3 ± 7.1	96.7 ± 9*	222.1 ± 10	0.0 ± 0.0	0.0 ± 0.0	0.3 ± 0.1	36.2 ± 3.2	0.2 ± 0.0	388 ± 44	1347 ± 78	2.0 ± 0.3	221 ± 53 *	2410 ± 170**	1349 ± 55	1242 ± 64
PUCALA x CATONGO	Control	1.8 ± 0.1*	21.6 ± 2*	1.6 ± 0.3*	65.4 ± 2**	0.0 ± 0.0	70.2 ± 3.8***	53.6 ± 5	197.1 ± 16***	0.0 ± 0.0*	0.0 ± 0.0	0.4 ± 0.0*	43.5 ± 4.3	0.3 ± 0.0	296 ± 16*	711 ± 62**	2.3 ± 0.1	143.5 ± 21*	1566 ± 124	952 ± 75*	820 ± 40**
	Drought	1.3 ± 0.0	16.4 ± 1	0.6 ± 0.1	28.7 ± 3	0.0 ± 0.0	31.8 ± 4.0	39.4 ± 6	100.9 ± 7	0.0 ± 0.0	0.0 ± 0.0	0.3 ± 0.0	29.3 ± 5.5	0.3 ± 0.0	219 ± 24	380 ± 30	2.1 ± 0.2	74.2 ± 3.4	1347 ± 146	468 ± 26	452 ± 35
CATONGO x TSH 1188	Control	1.8 ± 0.0	17.1 ± 0	1.1 ± 0.1	52 ± 3	0.0 ± 0.0	50.5 ± 3.4	46.1 ± 6	131.3 ± 10	0.0 ± 0.0	0.0 ± 0.0	0.4 ± 0.0	30.3 ± 1.8	0.3 ± 0.0	323 ± 44	695 ± 25	2.4 ± 0.1*	157.9 ± 24	1423 ± 123	853 ± 55	810 ± 77
	Drought	1.8 ± 0.0	17.8 ± 1	1.1 ± 0.0	47.6 ± 4	0.0 ± 0.0	58.0 ± 4.8	63.9 ± 5	148.6 ± 9	0.0 ± 0.0	0.0 ± 0.0	0.4 ± 0.0	27.4 ± 2.2	0.2 ± 0.0	319 ± 38	585 ± 46	1.8 ± 0.1	87.6 ± 23	1746 ± 24	879 ± 84	603 ± 67
RB 40 x CATONGO	Control	1.9 ± 0.1	20.0 ± 1	0.9 ± 0.0	59 ± 2	0.0 ± 0.0	55 ± 4.1	65 ± 4	157.2 ± 11	0.0 ± 0.0	0.0 ± 0.0	0.4 ± 0.0***	31.3 ± 1.8	0.2 ± 0.0	399 ± 27	1231 ± 70*	3.0 ± 0.1	324 ± 37*	1492 ± 71	1118 ± 71	1420 ± 160
	Drought	1.8 ± 0.1	22.0 ± 2	0.8 ± 0.1	41.4 ± 6	0.0 ± 0.0	49.7 ± 4.6	71.2 ± 5	172.7 ± 26	0.0 ± 0.0	0.0 ± 0.0	0.3 ± 0.0	33.9 ± 2.16	0.3 ± 0.0	350 ± 28	727 ± 72	1.9 ± 0.2	130 ± 12	1778 ± 83	927 ± 83	900 ± 55
MOC 01 x CATONGO	Control	1.9 ± 0.1**	24.5 ± 1	1.4 ± 0.1*	58 ± 2	0.0 ± 0.0	86.7 ± 5.6*	92.2 ± 9	224 ± 5*	0.0 ± 0.0	0.0 ± 0.0	0.4 ± 0.0	44.8 ± 4.1	0.2 ± 0.0	333 ± 36	945 ± 80	2.7 ± 0.1*	216 ± 51	1434 ± 117	743 ± 63	1041 ± 54
	Drought	1.4 ± 0.0	21.8 ± 2	0.9 ± 0.1	50.2 ± 6	0.0 ± 0.0	70.3 ± 1.9	75.4 ± 10	202.5 ± 15	0.0 ± 0.0	0.0 ± 0.0	0.3 ± 0.0	52.8 ± 5.2	0.2 ± 0.1	301 ± 66	586± 36	1.6 ± 0.4	91 ± 11	1889 ± 124	942 ± 88	780 ± 77
CATONGO x IMC 67	Control	1.9 ± 0.1	19.7 ± 2	1.3 ± 0.1	66.3 ± 3	0.0 ± 0.0	79.8 ± 6.1	71.8 ± 5	224.8 ± 21	0.0 ± 0.0	0.0 ± 0.0	0.4 ± 0.1	42.5 ± 4.8	0.2 ± 0.0	405 ± 29*	1620 ± 49*	3.6 ± 0.2**	458 ± 50 *	1489 ± 113	1000 ± 76	1558 ± 76
	Drought	1.9 ± 0.2	20.8 ± 2	1.1 ± 0.1	53.7 ± 5	0.0 ± 0.0	71.3 ± 7.1	96.7 ± 9*	222.1 ± 10	0.0 ± 0.0	0.0 ± 0.0	0.3 ± 0.1	36.2 ± 3.2	0.2 ± 0.0	388 ± 44	1347 ± 78	2.0 ± 0.3	221 ± 53 *	2410 ± 170**	1349 ± 55	1242 ± 64
PUCALA x TSH 1188	Control	1.7 ± 0.1	21.8 ± 1**	1.1 ± 0.1*	61.8± 5*	0.0 ± 0.0	44.8 ± 2.8	66.0 ± 6	136.1 ± 6	0.0 ± 0.0**	0.0 ± 0.0*	0.3 ± 0.0	32.5 ± 4.6	0.2 ± 0.0	235 ± 32	542 ± 29	2.8 ± 0.2*	181 ± 41	1238 ± 151**	584 ± 36	748 ± 65
	Drought	1.8 ± 0.1	16.4 ±1	0.7 ± 0.1	45.3 ± 4	0.0 ± 0.0	41.7 ± 3.8	56.5 ± 8	142.6 ± 7	0.0 ± 0.0	0.0 ± 0.0	0.4 ± 0.0	27.4± 2.3	0.3 ± 0.0	450 ± 39**	724 ± 57	1.9 ± 0.1	137 ± 26	1889 ± 124**	1291 ± 77**	971 ± 33
PUCALA x RB 40	Control	1.5 ± 0.1	19.0 ± 2	0.8 ± 0.1	45 ± 5*	0.0 ± 0.0*	48.2 ± 4.8	41.9 ± 5	115.13 ± 12	0.0 ± 0.0*	0.0 ± 0.0	0.4 ± 0.0	25.8 ± 3.2	0.3 ± 0.0	280 ± 19	625 ± 45	2.2 ± 0.1	110 ± 9.4	1360 ± 115	871 ± 84	726 ± 51
	Drought	1.8 ± 0.1	20.5 ± 1	0.8 ± 0.0	34.1 ± 2	0.0 ± 0.0	52.01 ± 1.5	66.0 ± 7*	152.2 ±11*	0.0 ± 0.0	0.0 ± 0.0	0.3 ± 0.0	31.9 ± 2.9	0.3 ± 0.0	391 ± 36*	949 ± 86	2.1 ± 0.2	181 ± 21	1902 ± 117*	1038 ± 66	1049 ± 95*
PUCALA x MOC 01	Control	1.8 ± 0.0	19.5 ± 1	1.4 ± 0.2*	64.3 ± 3*	0.0 ± 0.0	66.7 ± 6.8	62.4 ± 8	173.4 ± 16	0.0 ± 0.0	0.0 ± 0.0	0.4 ± 0.0**	28.3 ± 2.4	0.2 ± 0.0	364 ± 18	987 ± 45*	2.3 ± 0.1**	192.6 ± 30*	1756 ± 104	1307 ± 70*	1331 ± 130**
	Drought	1.7 ± 0.1	20.8 ± 1	0.8 ± 0.0	39 ± 2	0.0 ± 0.0	51.6 ± 2.6	62.8 ± 6	152.2 ± 11	0.0 ± 0.0	0.0 ± 0.0	0.3 ± 0.0	32.8 ± 2.9	0.3 ± 0.0	333 ± 18	489 ± 33	1.5 ± 0.0	57.0 ± 5.6	2220 ± 37*	968 ± 65	502 ± 49
PUCALA x IMC 67	Control	1.5 ± 0.1	17.8 ± 1	0.7 ± 0.1	42.8 ± 6	0.0 ± 0.0	56.3 ± 4.1	37.2 ± 4	131.1 ± 10	0.0 ± 0.0	0.0 ± 0.0	0.4 ± 0.0	29.0 ± 1.8	0.3 ± 0.0	176 ± 12	362 ± 36	2.2 ± 0.3	86.7 ± 12	1269 ± 63	560 ± 72	812 ± 44
	Drought	1.4 ± 0.1	20.8 ± 2	0.5 ± 0.0	30 ± 1	0.0 ± 0.0	53.3 ± 4.2	36.9 ± 4	129.5 ±12*	0.0 ± 0.0	0.0 ± 0.0	0.4 ± 0.0	30.7 ± 4.0	0.3 ± 0.0	244 ± 27*	572 ± 35	1.9 ± 0.1	90.1 ± 14	1648 ± 99*	752 ± 45	628 ± 43
RB 40 x TSH 1188	Control	2.0 ± 0.1	21.1 ±1	1.2 ± 0.0	68.8 ± 5*	0.0 ± 0.0	71.7 ± 6.4	59.6 ± 3	167.2 ± 10	0.0 ± 0.0*	0.0 ± 0.0	0.4 ± 0.0	31.4 ± 2.6	0.2 ± 0.0	372 ± 11	560 ± 50	2.1 ± 0.1*	160 ± 36	1790 ± 34	1275 ± 63**	1144 ± 54*
	Drought	2.0 ± 0.0	20.7 ± 1	0.9 ± 0.1	52.4 ± 2	0.0 ± 0.0	59.0 ± 5.0	74.8 ± 5	167.3 ± 9	0.0 ± 0.0	0.0 ± 0.0	0.3 ± 0.0	29.6 ± 2.2	0.2 ± 0.0	281 ± 14	431 ± 33	1.5 ± 0.1	52 ± 9.7	1718 ± 89	663 ± 34	375 ± 32
MOC 01 x TSH 1188	Control	1.9 ± 0.0**	21.1 ± 2*	1.3 ± 0.1	82.3 ± 4*	0.0 ± 0.0	70.1 ± 4.4**	81.1 ± 6	181.3 ± 12*	0.0 ± 0.0	0.0 ± 0.0	0.4 ± 0.0	34.1 ± 2.8	0.2 ± 0.0	390 ± 18	1006 ± 68	2.7 ± 0.2*	228 ± 35*	1468 ± 94	922 ± 42	1182 ± 67
	Drought	1.4 ± 0.1	17.6 ± 1	3.3 ± 0.0	64.5 ± 4	0.0 ± 0.0	47.5 ± 5.0	61.8 ± 5	130.8 ± 11	0.0 ± 0.0	0.0 ± 0.0	0.3 ± 0.0	31.7 ± 3.3	0.3 ± 0.0*	330 ± 8	769 ± 32	1.9 ± 0.1	86 ± 6	1823 ± 68	1017 ± 87	885 ± 56
IMC 67 x TSH 1188	Control	2.0 ± 0.1	21.8 ± 1*	1.4 ± 0.1***	76.8 ± 4***	0.0 ± 0.0	73.6 ± 6.9*	93.3 ± 7**	185.5 ± 14*	0.0 ± 0.0*	0.0 ± 0.0	0.4 ± 0.0	39.4 ± 3.2**	0.2 ± 0.0	414 ± 38*	1163 ± 54***	2.8 ± 0.1***	294 ± 27***	1878 ± 170	1101 ± 45*	1415 ± 146***
	Drought	1.8 ± 0.0	18.3 ± 1	0.9 ± 0.1	60.8 ± 2	0.0 ± 0.0	51.10 ± 4.4	60.6 ± 7	152.8 ± 4	0.0 ± 0.0	0.0 ± 0.0	0.4 ± 0.0	27.0 ± 1.57	0.3 ± 0.0	324 ± 13	529 ± 51	1.6 ± 0.0	69 ± 9	1956 ± 99	795 ± 62	489 ± 70
RB 40 x MOC 01	Control	2.0 ± 0.1	23.7 ± 2	1.1 ± 0.2	47.6 ± 2*	0.0 ± 0.0	64.7 ± 8.3	69.2 ± 9	161.0 ± 22	0.0 ± 0.0*	0.0 ± 0.0	0.4 ± 0.0***	22.7 ± 1.8	0.2 ± 0.0	441 ± 50*	1478 ± 60**	2.7 ± 0.1	262 ± 44	1833 ± 133	1009 ± 43	1438 ± 46*
	Drought	2.1 ± 0.1	24.2 ± 1	0.7 ± 0.1	36.2 ± 3	0.0 ± 0.0	52.7 ± 6.1	84.9 ± 8	183.08 ± 19	0.0 ± 0.0	0.0 ± 0.0	0.3 ± 0.0	38.6 ± 3.2**	0.3 ± 0.0**	308 ± 7.8	770 ± 66	2.7 ± 0.3	172 ± 54	1604 ± 190	856 ± 40	687 ± 35
RB40 x IMC 67	Control	2.1 ± 0.1*	20.6 ± 1	1.0 ± 0.0	65 ± 6*	0.0 ± 0.0	62.5 ± 4.7	65.3 ± 7	159.3 ± 14	0.0 ± 0.0	0.0 ± 0.0	0.4 ± 0.0	29.1 ± 2.5	0.2 ± 0.0	340 ± 45	1277 ± 35*	3.3 ± 0.4*	364 ± 43***	1473 ± 100*	743 ± 39*	1169 ± 109
	Drought	1.8 ± 0.0	18.7 ± 0	1.0 ± 0.1	47 ± 3	0.0 ± 0.0	53.9 ± 6.0	65.9 ± 6	143.1 ± 11	0.0 ± 0.0	0.0 ± 0.0	0.4 ± 0.0	36.3 ± 2.7	0.3 ± 0.1	394 ± 55	767 ± 43	1.8 ± 0.2	69 ± 9.0	2027 ± 154	1258 ± 88	784 ± 45
MOC 01 x IMC 67	Control	2.0 ± 0.1	21.6 ± 1.6*	1.1 ± 0.1	59.5 ± 4	0.0 ± 0.0	62.11 ± 6.6	67.3 ± 10	179.6 ± 26	0.0 ± 0.0	0.0 ± 0.0	0.3 ± 0.0	32.1 ± 2.67	0.2 ± 0.0	378 ± 19*	1149 ± 54**	2.9 ± 0.2***	308.3 ± 44***	1602 ± 109	1004 ± 71	1216 ± 108**
	Drought	1.9 ± 0.1	17.7 ± 0.6	0.8 ± 0.0	46.5± 4	0.0 ± 0.0	49.9 ± 4.8	62.2 ± 6	140.4 ± 11	0.0 ± 0.0	0.0 ± 0.0	0.3 ± 0.0	28.7 ± 2.2	0.2 ± 0.0	315 ± 18	524 ± 35	1.6 ± 0.0	70.6 ± 11.1	1755 ± 102	864 ± 55	518 ± 60
Mean	Control	1,9	21,4	1,2	62,0	0,0	66,3	67,6	174,0	0,0	0,0	0,4	34,0	0,2	349,0	966,3	2,6	233,6	1567,8	969,9	1137,9
	Drought	1,8	19,9	0,9	46,6	0,0	53,3	67,1	159,1	0,0	0,0	0,3	32,8	0,3	341,0	660,8	1,8	104,4	1876,5	938,9	707,5

H, high; CD stem diameter; TLA, total leaf area; NL, leaves number; ILA, individual leaf area; LDB, leaf dry biomass; SDB, stem dry biomass; TDB, total dry biomass; LAR, leaf area ratio; SLA, specific leaf area; LMR, leaf mass ratio; RDB, root dry weight; R/S, root/shoot ratio; RL, root length; RA, root area; RD, average root diameter; RV, root volume; RD, fine root diameter (< 1 mm), medium (1 < RD < 2 mm) and coarse (< 2 mm).).

*** Significance by the T test at p < 0.001

** p < 0.01

* p < 0.05.

The increase in RDB, under water stress, can be observed on most progenies (Table 4). This trait has been identified as one of the adaptive mechanisms of plants to drought tolerance. Mean increases in RDB, compared to the overall mean, were more expressive in MOC-01 x Catongo (61%), Catongo x IMC-67 (18%), Catongo x SCA-6 (12%), SCA-6 x IMC-67 (10%), RB-40 x IMC-67 (10%) and RB-40 x Catongo (3%). In contrast, for the same morphological trait, progenies of PUCALA x SCA-6, PUCALA x TSH-1188, Catongo x TSH-1188, MOC-01 x SCA-6, MOC-01 x IMC-67, IMC-67 x TSH-1188 and PUCALA x Catongo showed decreases of 24, 17, 16, 16, 14, 12 and 11%, respectively, from the overall mean of the progenies.

The expansion of the root system under water stress conditions was associated with the growth of fine (RD < 1 mm) and coarse (RD > 2 mm) roots. For the progenies of SCA-6 x IMC-67 (62%), MOC-01 x SCA-6 (42%) RB-40 x IMC-67 (38%), PUCALA x SCA-6 (33%), MOC-01 x Catongo (32%) PUCALA x IMC-67 (30%) RB-40 x Catongo (19%) RB-40 x MOC-01 (12%) and Catongo x SCA-6 (11%), there was an increase of RDB under water stress mainly due to the development of fine roots (Table 4). In contrast, Catongo x IMC-67, PUCALA x Catongo and RB-40 x SCA-6 showed reduced root growth under water stress, with values 20, 14 and 7%, lower, respectively, for fine roots, and 52, 45 and 45% lower for coarse roots, respectively. Progenies of PUCALA x MOC-01, IMC-67 x TSH-1188, MOC-01 x IMC-67 and PUCALA x SCA-6 and PUCALA x Catongo showed significant reductions in coarse root growth under water stress, with values of 62, 65, 57, 57 and 45% lower, respectively.

Under control condition, most of progenies showed mean values above the overall mean for more than 60% of the analyzed variables, the values were associated with morphological attributes of root development. In contrast, under drought, the progenies with higher means showed balance between the shoot and root system development.

In progenies identified as with high vegetative vigor, the mean values for morphological traits were higher than the overall mean of each variable analyzed (Table 4). With this, under the control condition, the following progenies were highlighted (variables and percentage above the general mean): Catongo x SCA-6 (+ 40% RDB, +33% RD and +90% RV), TSH-1188 x SCA (+40% TLA, +33% NL and +90% RA), RB-40 x SCA-6 (+44% RA and +97% VR), MOC-01 x SCA-6 (+64% RA, +52% RD and +210% RV), SCA-6 x IMC-67 (+145% RA, +93% RD and +339% RV), Catongo x IMC-67 (+124% RA, +47% RD and +151% RV), PUCALA x MOC-01 (+49% AR, +85% RV and +67% RD > 2 mm), IMC-67 x TSH-1188 (+76% RA, +52% RD, +182% RV and +77% RD > 2 mm) and RB-40 x MOC-01 (+40% RA and +59% RV).

For progenies of low vegetative vigor, the means shown were lower than the overall mean of each variable analyzed, with negative results for: PUCALA x SCA-6 (-18% TLA, -20% NL and -27% RD <1 mm), Catongo x TSH-1188 (-20% CD, -24% LDB and -32% SLD), RB-40 x Catongo (-22% TLA, -18% IAL and 20% RD < 2 mm), PUCALA x TSH-1188 (-21% IAL, -32% LDB and -38% 1 mm < RD < 2 mm), PUCALA x RB-40 (-20% H, 35% TLA and -28% RD <1 mm) and PUCALA x IMC-67 (-22% H, -38% TLA and -40% RD > 2 mm) (Table 4).

Analyzing the established criteria for drought tolerance, it can be observed that for some progenies, the means values were lower than the overall mean of each growth variable with negative results for: PUCALA x 6 SCA (-30% LDB, -23% SDB and -43% RV), PUCALA x Catongo (-30% TLA, -40% LDB and -51% RD > 2 mm), PUCALA x MOC-01 (-16% LN, -26% RA and -37% RD > 2 mm), IMC-67 x TSH-1188 (-21% ILA, -20% RA and -39% RD > 2 mm) and MOC-01 x IMC-67 (-21% RA, -32% RV and -35% RD > 2 mm (Table 4). On the other hand, the progenies Catongo x SCA-6 (+18% AFT, +22% RV and +14% SDB), SCA-6 x IMC-67 (+15% H, +22% CD, +25% TLA and +56% RD > 2 mm), MOC-01 x Catongo (+32% LDB, +27% TDB and +61% RDB), Catongo x IMC-67 (+26% SDB, +21% R/S and +65% RV), RB-40 x MOC-01 (+12% CD, +33% LDB and +22% RDB) and RB-40 x IMC-67 (+15% LDB, +16% RA and +34% RD > 2 mm) showed mean values above the general mean in most variables analyzed.

Breaking the sum of squares of the progenies into sum of squares for general (GCA) and specific (SCA) combining abilities significant effects for the analyzed growth variables were also observed (Table 5). This indicates that additive and non-additive effects are involved in the genetic control of these traits. For TLA and R/S only significant effects were detected for SCA, unlike LAR, where only a significant effect on GCA was found.

**Table 5:** Estimates of the specific and general combining ability (SCA and CGA, respectively) for root and shoot morphological attributes, used in the analysis of contrasting cacao genotypes for drought, submitted for 60 days to two water conditions (SI): control (Ψ_WL_ between -0.1 and -0.5 MPa) and stressed (Ψ_WL_between -2.0 and -2.5 MPa). Ilhéus, Bahia, Brazil.

Source of Variation	DF										Mean Squares										
		H	CD	TLA	LN	ILA	LDB	SDB	TDB	LAR	SLA	LMR	RDB	R/S	RL	RA	RD	RV	RD < 1	1< RD < 2	RD > 2
		(m)	(cm)	(m^2^)	(unit.)	m^2^.plant^-1^	g.plant^-1^	g.plant^-1^	g.plant^-1^	(m^2^. g^-1^)	(m^2^. g^-1^)	(g/g)	g.plant^-1^		cm	cm^2^	mm	cm^3^	mm	mm	mm
PROG	20	0.3***	36.4***	25922308.5***	1241.2***	7094.0***	1124.2***	2033.8***	7787.9***	609.7***	2438.6***	0.1***	311.1***	0.1***	2740921.0***	662209.0***	0.8***	41602.0***	316373.0**	229150.0***	505578.0***
CGA	6	8.2***	54.3***	50184884.7**	3153.9***	12650.9**	2051.8***	3832.6***	12940.5***	373.6^ns^	4500.3***	0.1*	326.2***	0.2***	4656275.0***	999515.0***	0.9**	67516.0***	360036.0**	245226.0*	523232.0**
SCA	14	0.9**	2878.0**	15524061.1^ns^	421.4**	4711.8**	726.7***	1262.9**	5581.1**	711.0 ***	1555.1**	0.1***	304.7***	0.0^ns^	1920055.0*	517649.0**	0.7**	30497.0**	297660.0*	222260.0**	498013.0**
SI	1	0.5**	134.6***	73566219.1***	1472.5***	145.37^ns^	10764.0**	23.4^ns^	13792.4**	1517***	13793.3***	0.2***	75.2^ns^	0.1***	1296967.0^ns^	5775153.0***	37.2***	1050501.0***	4960373.0***	108150.0^ns^	12021085.0***
PROG x SI	20	0.1**	19.3*	25771937.7**	152.0^ns^	3476.0**	417.1**	821.6**	2550.4*	434.9**	1552.6***	0.1^ns^	163.2**	0.1**	2026416.0**	234308.0**	0.6**	25034.0**	325898.0**	310693.0***	315837.0*
CGA x SI	6	0.1^ns^	10.5^ns^	23284304.3*	110.6^ns^	5006.3**	370.7^ns^	933.5**	1913.5^ns^	233.8^ns^	1792.7**	0.1^ns^	126.2^ns^	0.0^ns^	2492091.0*	312912.0*	0.7*	44276.0***	199644.0^ns^	120041.0^ns^	280235.0^ns^
SCA x SI	14	0.1**	23.0*	26838066.3 **	170.5^ns^	2820.6*	436.9 **	774.9***	2823.3 *	521.1**	1449.8**	0.1^ns^	179.1**	0.0***	1826840.0*	200620.0*	0.5*	16787.0^ns^	380006.0**	392401.0***	331094.0*
Residual	248	0.0	10.9	10427373.0	115.0	1621.5	181.1	353.1	1522.0	219.5	569.4	0.1	72.4	0.0	884697.0	116302.5	0.2	10905.1	142165.0	95446.1	180697.0

H, high; CD stem diameter; TLA, total leaf area; NL, leaves number; ILA, individual leaf area; LDB, leaf dry biomass; SDB, stem dry biomass; TDB, total dry biomass; LAR, leaf area ratio; SLA, specific leaf area; LMR, leaf mass ratio; RDB, root dry weight; R/S, root/shoot ratio; RL, root length; RA, root area; RD, average root diameter; RV, root volume; RD, fine root diameter (RD < 1 mm), medium (1 < RD < 2 mm) and coarse (< 2 mm).

*** significance by F test at p < 0.001

** p < 0.01

* p < 0.05.

The results of the joint diallel analysis revealed significant effects (p < 0.05) between GCA x SI and between SCA x SI for most characters. For TLA, ILA, SDB, SLA, RL, RA and RD the effects of SCA and GCA were highly significant. However, their estimates varied depending on environmental stimuli, since the interaction of these parameters with the water regime (SCA x SI and GCA x SI) were also significant. However, the variance component of the interaction GCA x SI was higher than the variance component of the SCA x SI interaction for the variables: RA (56%), ILA (44%), RD (41%), RL (36%), SLA (19%) and SDB (17%) (Table 5). In contrast, for H, CD, LDB, TDB, LAR, RDB, R/S, RD < 1 mm, 1 < RD < 2 mm and RD> 2 mm a significant (p <0.05) effect was only observed in the interaction SCA x SI. These findings allow inferring that there were differential responses of the hybrid combinations as a function of water regimes, and existence of little variation among parents.

The effects of general combining ability of the seven parents are shown in Table 6. As there were different responses of the parents in the tested water regimens, separate analyses of combining abilities were performed. In the control condition, the parents SCA-6, RB-40 and MOC-01 showed positive values for H and CD, while PUCALA showed negative values for these characters. Under water stress, only SCA-6 and RB-40 showed positive values for H (0.3 and 0.1) and CD (0.9 and 1.3, respectively.

**Table 6:** Estimates of the combining capacity effects (GCA) for seven contrasting cacao genotypes for drought submitted for 60 days to two water conditions (SI): control (Ψ_WL_ between -0, 1 and -0.5 MPa) and drought (Ψ_WL_ between -2.0 to -2.5 MPa). Ilhéus, Bahia, Brazil.

Parents											General Combining Ability (g_i_)									
	H	CD	TLA	LN	ILA	LDB	SDB	TDB	LAR	SLA	LMR	RDB	R/S	RL	RA	RD	RV	RD < 1	1< RD < 2	RD > 2
												Control								
SCA 06	0.1	1.8	1192.0	4.9	6.7	10.1	7.6	20.3	-0.9	-3.4	-0.1	1.7	-0.2	200.1	178.5	0.1	68.6	-4.5	39.8	172.1
CATONGO	-0.0	0.0	458.4	-5.9	15.5	2.4	-0.5	8.3	-4.6	-0.0	0.2	-0.1	0.0	76.8	34.5	-0.0	-26.1	-2.8	-40.8	-55.8
PUCALA	-0.2	-1.3	-1142.7	-9.0	5.3	-10.2	-15.3	-23.8	1.2	-4.8	0.2	-1.9	0.2	-994.2	-345.1	-0.2	-99.9	-164.9	-140.0	-272.7
TSH 1188	0.0	-0.9	1218.9	11.3	-19.5	-5.4	1.0	-15.8	3.7	14.9	-0.1	-1.2	0.0	103.6	-118.1	-0.3	-5.4	3.3	29.3	17.7
RB 40	0.0	0.3	-1731.7	-2.9	-9.2	-1.1	-3.0	-8.6	-1.7	-11.6	0.1	0.1	0.0	141.9	-50.6	-0.1	-17.1	87.7	119.0	-105.5
MOC 01	0.0	0.7	1042.4	3.3	-4.6	5.0	11.0	19.8	2.7	0.2	0.0	3.3	0.1	359.2	54.4	-0.0	6.2	38.3	73.1	78.0
IMC 67	0.0	-0.5	-1037.4	-1.7	5.7	-0.9	-0.7	-0.3	-0.3	4.7	0.0	-1.9	-0.1	112.3	246.3	0.3	73.8	43.0	-80.5	166.1
												Drought								
SCA 06	0.2	0.9	348.9	4.5	-10.5	1.3	5.6	16.8	-0.9	-8.3	-0.1	-1.3	-0.3	115.3	91.9	-0.0	16.2	71.2	23.6	71.3
CATONGO	-0.0	0.1	168.3	-4.8	16.8	-0.4	1.7	4.5	2.6	4.6	-0.1	4.3	0.2	-266.8	-49.2	0.2	10.9	-218.1	-124.6	-4.3
PUCALA	-0.2	-1.0	-1959.4	-14.0	12.2	-10.3	-17.9	-25.4	-4.8	-21.1	0.2	-3.9	0.2	-124.4	-83.5	0.0	-5.9	-36.9	-37.0	-35.8
TSH 1188	-0.0	-1.3	52.7	9.3	-38.5	-4.9	-4.1	-11.1	3.8	11.3	-0.1	-4.1	-0.1	110.0	-20.1	-0.0	-8.8	-20.2	26.3	-3.9
RB 40	0.1	1.3	622.8	-0.2	25.3	5.8	12.2	10.1	-3.6	2.1	-0.1	1.5	-0.1	139.9	0.9	-0.1	-3.0	34.1	35.3	-12.3
MOC 01	-0.0	-0.4	849.7	5.5	-1.8	6.2	1.4	1.2	3.5	12.9	0.1	3.1	0.1	225.8	-40.3	-0.1	-22.1	127.4	16.0	-36.7
IMC 67	0.0	-0.1	-83.1	-0.3	-3.5	2.3	0.8	3.7	-0.5	-1.6	0.1	0.3	-0.1	-199.8	101.2	0.0	12.7	42.4	60.2	21.9

H, high; CD stem diameter; TLA, total leaf area; LN, leaf number; ILA, individual leaf area; LDB, leaf dry biomass; SDB, stem dry biomass; TDB, total dry biomass; LAR, leaf area ratio; SLA, specific leaf area and MRL, leaf mass ratio; RDB, root dry weight; R/S, root/shoot ratio; RL, root length; RA, root area; RD, average root diameter; RV, root volume; RD, fine root diameter (RD < 1 mm), medium (1 < RD < 2 mm) and coarse (< 2 mm).

For TLA, in the control condition, SCA-6, Catongo, TSH-1188 and MOC-01 showed positive values for GCA, while in PUCALA, RB-40 and IMC-67 were negative. The parents SCA-6 (348.9), Catongo (168.4), TSH-1188 (52.7), RB-40 (622.8) and MOC-01 (849.8) formed the group with high GCA for leaf area increase under drought condition, while PUCALA (-1959.4) and IMC-67 (-83.2) showed low GCA for shoot development (Table 6).

SCA-6, TSH-1188 and MOC-01 were the parents that showed positive values for stem development under control conditions with GCA values of 8, 1 and 11, respectively. While under water deficit, only PUCALA and TSH-1188 showed negative GCA values for biomass allocation to the stem as an adaptive plant response to stress (Table 6).

For RDB, in the control condition, SCA-6, RB-40 and MOC-01 showed positive values for GCA (1.7, 0.2 and 1.4, respectively). While Catongo, PUCALA, TSH-1188 and IMC-67, showed low GCA for RDB, with values of -0.2, -1.9, -1.2 and -1.9, respectively. Under water stress, only Catongo, RB-40, MOC-01 and IMC-67 showed positive values of GCA for expansion of the root system in their crosses (Table 6).

In the control condition, RB-40 and MOC-01 showed high GCA for root development associated with fine (RD < 1 mm) and medium (1 mm <RD < 2 mm) roots, while SCA-6 showed high GCA for medium (1 mm < RD < 2 mm) and coarse (RD> 2 mm) roots. In contrast, under drought, the parents RB-40, MOC-01 and IMC-67 showed high GCA for the root system development, mainly associated with fine (RD <1 mm) and medium (1 mm <RD <2 mm) roots.

The estimated specific combining ability (s_ij_) values, evaluated based on the variables that showed significant effect (p < 0.05) of the SCA x A interaction are shown in Table 7. The progenies MOC-01 x IMC-67, PUCALA x MOC-01, Catongo x IMC-67, MOC-01 x Catongo, SCA-6 x IMC-67, Catongo x SCA-6, MOC-01 x SCA-6, PUCALA x TSH-1188, PUCALA x IMC-67, RB-40 x MOC-01 and MOC-01 x IMC-67 showed high SCA (negative) for shoot traits under control conditions, with increase of H, CD, LN and TLA, and intermediate development for the root system in the control condition. In contrast, under the same conditions RB-40 x IMC-67 and RB-40 x SCA-6 were the progeny with low SCA (positive) for the same attributes.

**Table 7:** Estimates of the specific combining ability (SGA) for seven contrasting cacao genotypes for drought submitted conditions control (Ψ_WL_ between -0.1 and -0.5 MPa) and stressed (Ψ_WL_ between -2.0 to -2.5 MPa),respectively. Ilhéus, Bahia, Brazil.

PROG	H	CD	TLA	LN	ILA	LDB	SDB	TDB	LAR	SLA	R/S	RDB	R/S	RL	RA	RD	RV	RD < 1	1< RD < 2	RD > 2
										Control										
1	0.0	1.8	-1.3	2.2	-24.0	6.4	3.1	-8.5	-12.4	-14.3	-0.1	-5.3	-0.2	-235.9	-252.9	-0.2	-78.6	-12.5	-28.2	-208.0
2	0.1	-0.3	-1.4	-7.5	12.7	-3.2	8.8	1.0	-11.4	-17.6	0.0	1.2	0.0	-300.8	62.4	-0.0	4.4	-52.8	-24.8	32.6
3	0.0	1.7	2.4	4.0	11.9	-5.7	-9.8	-15.5	3.6	13.5	0.0	-3.9	0.0	551.0	231.3	-0.0	57.8	139.9	193.8	316.3
4	-0.0	1.7	1.2	0.3	4.9	10.8	7.5	29.1	15.6	11.6	-0.0	4.0	-0.0	16.0	-147.4	-0.3	-79.9	264.0	-29.3	-369.9
5	-0.1	-2.6	-1.2	-0.6	9.0	-12.0	-6.9	-37.0	-1.5	11.4	0.0	-4.6	0.0	-248.8	-119.4	0.1	14.7	-205.4	-181.9	52.4
6	-0.0	1.2	0.4	1.5	-14.6	3.7	-2.8	30.8	6.1	-4.7	0.0	8.7	0.0	218.5	226.0	0.4	81.5	-133.0	70.6	176.7
7	0.1	1.6	4.4	13.9	21.8	11.1	1.6	38.6	6.9	4.5	0.0	11.6	-0.0	359.7	52.2	-0.0	19.0	149.8	162.9	5.4
8	-0.0	-3.2	-2.3	-15.0	6.1	-13.4	-22.1	-35.3	12.1	6.2	0.0	-2.2	0.0	-462.9	-191.0	-0.1	-44.5	-161.3	-105.6	-295.1
9	-0.0	-1.6	-1.4	6.2	-42.4	-12.7	0.8	-16.4	-4.7	9.6	-0.0	-2.6	0.0	258.2	277.8	0.5	121.4	-174.4	69.7	438.1
10	-0.0	2.4	0.5	-1.0	-1.1	12.3	13.9	42.5	-5.0	-4.7	0.0	7.6	0.0	-624.8	-113.5	0.1	2.1	-185.1	-259.3	-124.5
11	0.00	-1.0	0.2	-6.3	39.7	-3.7	2.5	-20.8	3.1	-1.4	0.0	-9.1	-0.0	705.6	227.4	-0.2	-19.4	383.7	160.5	184.1
12	-0.0	2.7	-1.2	-2.1	-26.6	-6.3	12.5	1.8	0.8	9.3	-0.1	1.6	-0.0	-275.4	35.1	0.5	52.3	-184.4	-275.5	-139.8
13	-0.1	-1.2	-1.4	-4.6	14.5	-7.1	-7.4	-26.3	-3.0	-4.2	0.0	-6.3	0.0	140.0	51.6	0.1	-6.8	-146.8	-77.6	-38.8
14	0.1	-1.2	-2.2	8.4	-24.5	5.0	-1.1	3.4	13.9	5.8	0.0	-7.1	-0.0	945.9	307.7	-0.0	52.3	427.5	404.0	382.3
15	-0.2	-1.5	-2.5	-8.0	2.0	0.6	-14.5	-18.5	-7.2	2.1	0.0	-1.0	0.0	-869.4	-509.2	-0.4	-121.2	-193.1	-188.9	-241.6
16	0.0	0.3	0.4	-1.2	11.4	11.4	-6.1	17.7	-4.0	-12.0	0.0	-1.4	-0.0	-45.1	-240.7	-0.3	-50.8	115.1	156.0	88.0
17	-0.0	0.0	-1.3	6.0	-24.5	3.5	1.1	3.3	-7.8	-7.6	0.0	-1.9	-0.1	-79.0	99.6	0.1	-6.3	-157.4	-150.7	-56.8
18	0.0	1.8	2.0	8.3	21.5	10.5	24.3	27.9	-4.7	-9.4	0.0	7.9	-0.0	311.4	65.4	-0.1	-8.3	248.1	181.9	87.4
19	-0.0	1.2	0.6	-9.0	50.6	-0.1	3.7	1.4	-2.9	-11.7	-0.0	9.4	0.01	1.8	-52.9	-0.2	-56.9	184.2	146.5	-82.0
20	0.2	-0.4	0.6	8.2	-39.1	-2.2	1.3	-5.5	-0.7	6.6	-0.1	-3.0	-0.0	-371.1	111.6	0.3	73.2	-242.1	-265.4	-35.2
21	0.0	0.1	-0.8	-3.7	-9.4	-8.8	-10.8	-13.7	3.4	6.8	-0.0	-3.3	0.0	4.9	-121.3	-0.0	-5.8	-63.6	41.3	-171.4
										Drought										
1	0.1	0.3	1.1	3.3	11.9	1.5	2.2	-10.9	6.0	1.0	0.0	1.0	0.0	113.6	-3.3	-0.12	-4.2	14.5	49.4	21.4
2	-0.0	-0.9	0.0	-6.1	15.1	-6.7	-3.2	-0.8	1.3	17.4	-0.0	-2.5	0.0	-116.5	-205.9	-0.3	-55.4	-83.2	-47.1	-283.8
3	-0.0	1.5	-3.8	-8.2	-10.1	-12.4	-4.1	-10.4	-19.4	-13.6	-0.1	3.9	0.1	142.0	102.6	0.4	40.7	-143.8	78.4	121.0
4	-0.0	-0.5	-0.1	4.7	-29.5	3.3	4.8	2.9	5.0	-4.4	0.0	0.3	-0.0	-687.6	-301.7	-0.2	-60.1	-165.0	-217.4	-313.4
5	0.1	-3.3	0.4	3.0	-10.1	-0.1	-22.6	-23.1	10.3	0.2	0.0	-7.0	-0.0	-45.9	-78.8	0.0	-8.5	-54.2	-199.5	12.1
6	0.0	2.9	1.9	3.2	22.7	14.4	23.0	42.4	-3.3	-0.6	0.0	4.2	-0.0	594.4	487.1	0.0	87.7	431.7	336.2	442.6
7	-0.1	-2.6	-0.8	1.3	-3.6	-10.6	-11.5	-37.2	5.0	-2.0	0.0	-3.9	0.0	-791.2	-153.5	-0.0	-35.8	-262.6	-298.8	-214.2
8	0.1	-0.8	2.2	-3.1	24.7	10.1	-0.8	-3.8	14.1	27.7	0.1	-5.6	-0.1	-26.1	-12.7	-0.3	-19.5	119.7	48.1	-94.6
9	-0.0	0.6	-1.2	0.2	-18.6	-8.9	-9.9	-1.0	-14.7	-7.6	-0.0	-4.9	0.0	254.0	109.4	-0.1	17.7	97.9	87.5	210.3
10	-0.2	2.1	-0.1	3.1	-18.9	11.1	5.0	37.5	-14.4	-29.7	0.0	12.3	0.0	278.1	8.9	0.0	-2.2	115.6	122.3	114.5
11	0.2	0.4	-1.0	-4.9	4.5	-3.2	15.0	15.6	3.8	10.6	-0.0	1.0	0.0	171.4	51.2	0.5	44.0	-85.1	-8.6	-37.4
12	0.2	-0.8	0.4	3.6	-17.1	3.7	11.3	20.1	-2.6	1.4	-0.0	2.6	-0.0	1143.1	160.6	0.0	47.5	263.4	374.2	304.1
13	0.0	0.4	0.7	2.1	15.0	3.1	4.5	8.4	5.1	11.6	-0.0	1.5	-0.0	519.1	365.8	0.4	85.3	39.9	111.2	390.3
14	0.1	2.3	0.8	1.1	15.8	2.3	12.1	17.3	-0.7	8.1	-0.0	0.7	0.0	-144.4	-53.8	-0.2	-19.8	264.6	60.2	-131.6
15	-0.2	1.6	-1.3	-2.0	-25.2	8.1	-13.1	-7.7	-8.2	-36.6	0.0	1.5	-0.0	-610.1	-113.2	-0.0	-21.7	-222.0	-199.7	-64.6
16	0.0	0.8	0.2	-3.0	13.3	4.7	-0.4	9.1	1.1	-16.1	0.0	-0.7	-0.0	-813.4	-266.2	-0.2	-41.0	-160.4	-327.1	-314.6
17	-0.3	-0.5	0.4	3.1	-10.7	-7.0	-2.6	-18.3	11.2	11.4	-0.0	-0.2	0.0	-403.5	234.9	0.4	12.3	-148.2	46.3	219.4
18	-0.0	-0.0	0.5	7.6	-0.0	0.7	-3.2	3.3	-4.5	-10.9	0.0	-0.0	0.0	-42.1	-219.3	-0.3	-40.0	69.4	-219.9	-235.3
19	0.1	1.4	-0.7	-5.4	22.9	5.7	15.4	10.4	-7.6	-5.6	0.0	2.3	0.0	290.1	93.5	0.1	43.3	101.8	112.1	-40.9
20	-0.2	-2.8	0.7	1.2	-3.1	-8.1	-14.3	-29.9	11.0	22.2	0.0	1.4	0.0	-110.8	-0.9	-0.0	-45.0	85.7	233.5	68.3
21	0.2	-2.1	-0.8	-5.0	1.0	-11.9	-7.2	-23.7	1.2	15.4	-0.0	-8.1	-0.0	-129.0	-204.8	-0.2	-25.0	-279.7	-141.4	-173.6

(1) CATONGO x SCA 6, (2) PUCALA x SCA 6, (3) TSH 1188 x SCA 6, (4) RB 40 x SCA 6, (5) MOC 01 x SCA 6, (6) SCA 6 x IMC 67, (7) PUCALA x CATONGO, (8) CATONGO x TSH 1188, (9) RB 40 x CATONGO, (10) MOC 01 x CATONGO, (11) CATONGO x IMC 67, (12) PUCALA x TSH 1188, (13) PUCALA x RB 40, (14) PUCALA x MOC 01, (15) PUCALA x IMC 67, (16) RB 40 x TSH 1188, (17) MOC 01 x TSH 1188, (18) IMC 67 x TSH 1188, (19) RB 40 x MOC 01, (20) RB 40 x IMC 67, (21) MOC 01 x IMC 67H, high; CD stem diameter; TLA, total leaf area; LN, leaf number; ILA, individual leaf area; LDB, leaf dry biomass; SDB, stem dry biomass; TDB, total dry biomass; LAR, leaf area ratio; SLA, specific leaf area and MRL, leaf mass ratio RDB, root dry weight; R/S, root/shoot ratio; RL, root length; RA, root area; RD, average root diameter; RV, root volume; RD, fine root diameter (RD < 1 mm), medium (1 < RD < 2 mm) and coarse (< 2 mm).

Under water limitation, progenies of Catongo x IMC-67, RB-40 x IMC-67, PUCALA x IMC-67, MOC-01 x SCA-6 and RB-40 x SCA-6 showed high SCA (negative) for shoot traits, in addition to an increased capacity to expand the root system. On the other hand, under the same conditions, TSH-1188 x SCA-6, PUCALA x MOC-01, PUCALA x TSH-1188, Catongo x TSH-1188 and RB-40 x Catongo were the progenies with lower SCA (positive) for the same attributes (Table 7).

**Fig 1:**
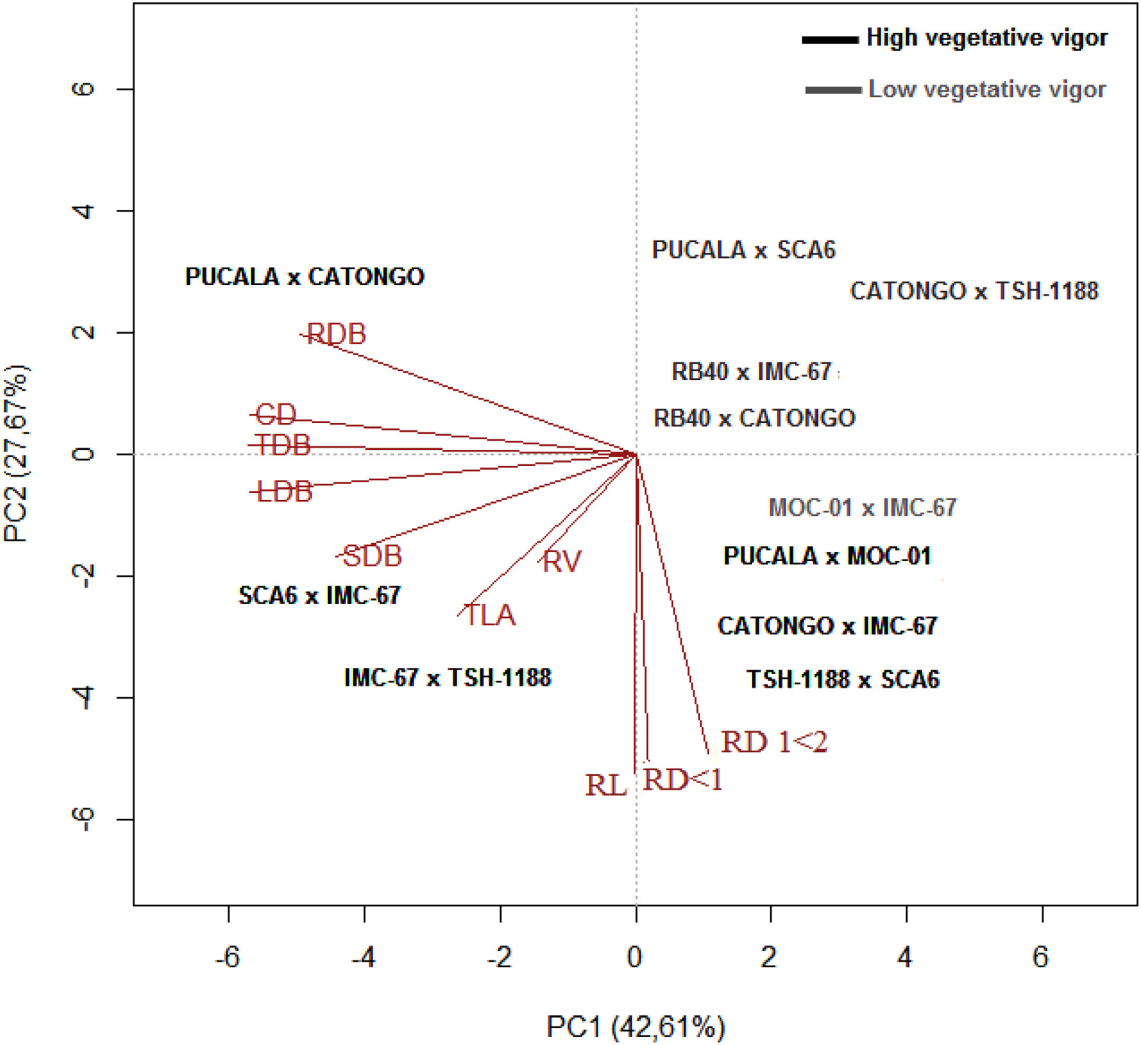
Biplot graphical analysis based on the mean of 21 cacao progenies under control condition (Ψ_WL_ between -0.1 and -0.5 MPa.) for CD, stem diameter; TLA, total leaf area; LDB, leaf dry biomass; SDB, stem dry biomass; TDB, total dry biomass; RDB, root dry weight; RL, root length; RV, root volume; RD, fine (RD <1 mm) and medium (1 < RD < 2 mm) root diameters.

The biplot analysis, based on main components, allowed separating genotypes based on the shown characteristics under the two water availability conditions. In the control condition (Ψ_WL_ between -0.1 and -0.5 MPa), the first and second principal component (CP1 and CP2) explained 43% and 27%, respectively, of the total variance, with a cumulative value of 70% (Fig 1). According to the relative length of each vector, the characteristic that most contributed to the total explained variance was TDB, followed by LDB, SDB and CD. Regarding the relationship between traits, there was a positive association between CD and RDB, LDB and SDB, RL with RD <1 mm and with 1 < RD <2 mm.

The dispersion analysis of the progenies identified that PUCALA x Catongo, SCA-6 x IMC-67, IMC-67 x TSH-1188 and MOC-01 x Catongo were the progenies that showed higher shoot development under control conditions. In contrast, under these conditions, PUCALA x MOC-01, Catongo x IMC-67 and TSH-1188 x SCA-6 performed better in morphological attributes of the root system. Furthermore, PUCALA x SCA-6, RB-40 x IMC-67, RB-40 x Catongo and Catongo x TSH-1188 were the progeny that had the lowest shoot and root development.

**Fig 2:**
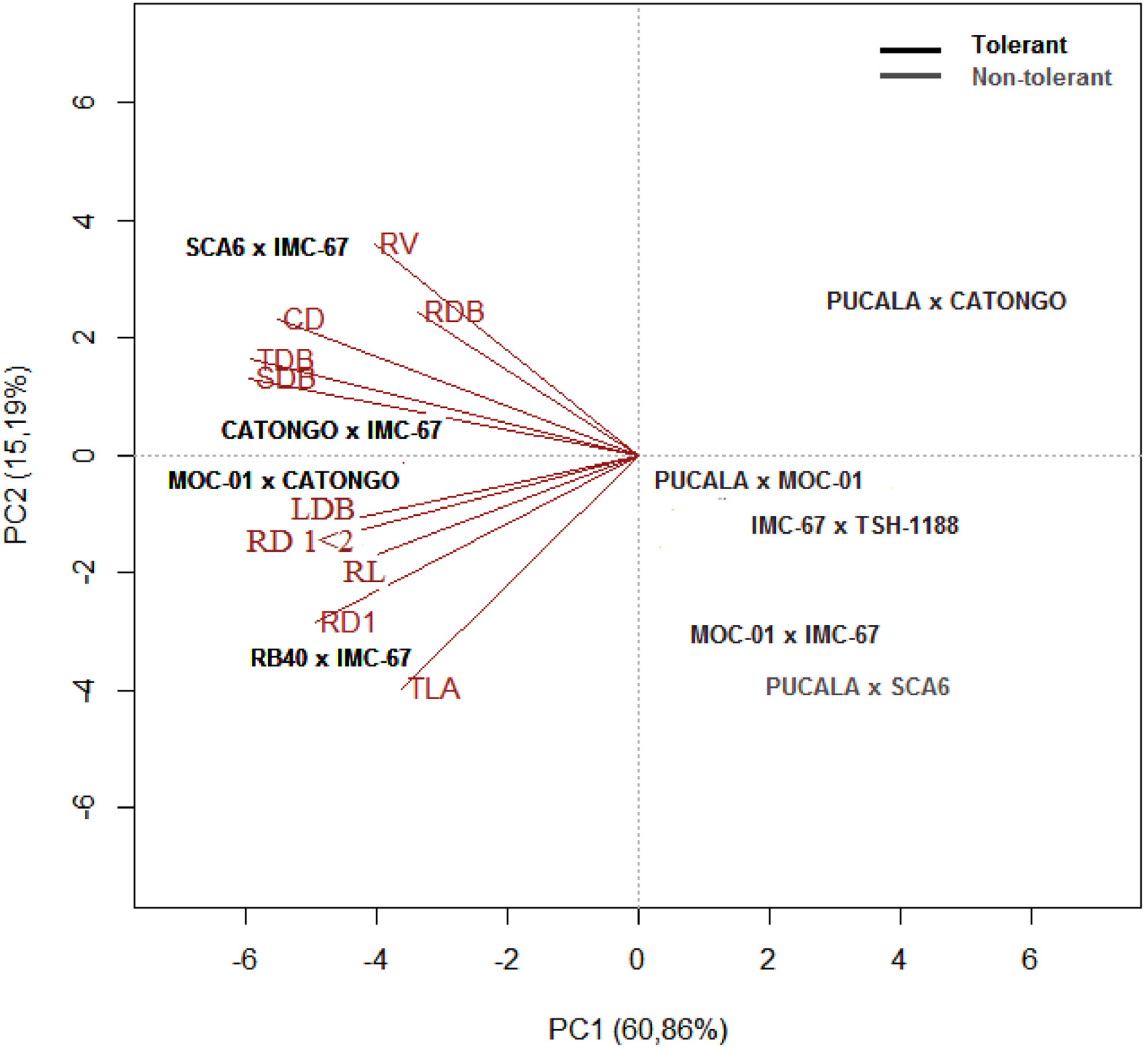
Biplot graphical analysis based on the mean of 21 cacao progenies under stress conditions (Ψ_WL_ between -2.0 and -2.5 MPa) for CD, stem diameter; TLA, total leaf area; LDB, leaf dry biomass; SDB, stem dry biomass; TDB, total dry biomass; RDB, root dry biomass; RL, root length; RV, root volume; RD, fine (RD < 1 mm) and medium (1 <RD <2 mm) root diameter.

Under limiting water conditions, the first and second principal components explained 63% and 15%, respectively, of the total variance, with a cumulative value of 78% (Fig 2). The trait that most contributed to the total explained variance was TDB, followed by SDB, RL and CD. Regarding the relationship between variables, there was a positive association between TDB and SDB, CD and LDB and RL with RD < 1 mm and 1 < RD < 2 mm.

In the analysis of dispersion, SCA-6 x IMC-67, IMC-67 x Catongo, MOC-01 x Catongo and RB-40 x IMC-67 were the progenies that showed greater root and shoot development under the stress condition; especially the last two progenies, which showed better results under limited soil water. Furthermore, RB-40 x Catongo, Catongo x TSH-1188 and TSH-1188 x SCA-6 showed intermediate development, with shoot reduction and root biomass increase. In contrast, PUCALA x Catongo, PUCALA x MOC-01, IMC-67 x TSH-1188 and MOC-01 x IMC-67 were the progeny that showed the least significant results regarding shoot and root development under limited water condition.

## Discussion

It was found that water deficit influenced biomass production, reducing the dry biomass of all plant organs in most of the evaluated *T*. *cacao* progenies. Less expressive effects were found in progenies of SCA-6 x IMC-67, MOC-01 x Catongo, Catongo IMC x 67 and RB-40 x IMC-67, which showed mean values above the general mean of the crosses for more than 60% of the analyzed variables and not significant reductions in dry biomass on water deficit. On the other hand, progenies of PUCALA x Catongo, PUCALA x MOC-01, IMC-67 x TSH-1188 and MOC-01 x IMC-67 proved to be non-tolerant to limitation of soil water with lower mean values than the overall mean of the crosses over 60% of the analyzed variables and significant reductions in dry biomass plant. These results corroborate studies with *Eucalyptus* sp, which identified variable resistance to soil water limitation as a function of different genetic material [24, 50, 51].

Non-tolerant progenies to soil water deficit decreased leaf biomass, with mean reductions, compared to the control, of -28, -24, -21 and -10% for TLA, LN, LAR and LMR, respectively (Table 4). Furthermore, comparing the mean of each progeny with the overall mean of any given trait, the results were even more expressive in progenies of PUCALA x SCA-6 (-30% LDB), PUCALA x Catongo (-30% TLA and -40% LDB) IMC-67 x TSH-1188 (-21% ILA), PUCALA IMC x 67 (-38% TLA and -35% LN). Leaf fall has been identified as an acclimation strategy to limited soil water in *Populus*, *Coffea* and *Eucalyptus* [52, 53, 54]. In *T*. *cacao* reduction in leaf area growth rate and total leaf area can be considered as one of the first responses to drought stress as a result of reduced cell turgor and net photosynthesis [55, 56, 57].

Progenies of Catongo x SCA-6 (+18% TLA), SCA-6 x IMC-67 (+25% TLA, 34% LDB and +40% TDB), MOC-01 x Catongo (+32% LDB and 27% TDB), RB-40 x MOC-01 (+33% LDB) and RB-40 x IMC-67 (+15% LDB) showed mean values above the general mean for some foliar biomass variables, indicating efficiency in carbon use as a response for acclimatization to water limitation in the soil. Considering that the stress can manifest itself in several degrees of severity, plants seek to optimize the source-sink relationships to keep partitioning assimilates and, at the same time, enabling responses of physiological and morphological acclimations to allow stability compatible with the moisture [51, 52, 58, 59, 60, 61, 62].

The drought not only limited the size of individual leaves, but also the number of leaves per plant, characteristic shown by the progenies PUCALA x Catongo and PUCALA x MOC-01, which had a reduction in LN of 39% and 16%, respectively. Plants under water stress can alter interception of solar radiation as one of the survival strategies [63]. Reduction of leaf number can contribute to water conservation by the plant, decreasing the transpiratory surface and the metabolic expenditure for maintenance of that tissue [51, 61]. In clones of *Coffea robusta*, grown under field conditions, leaf fall in response to water stress occurred sequentially: from older leaves to younger leaves, suggesting that the higher the sensitivity of the clone to drought, the greater the extent of leaf fall [54].

The growth variables H, CD and SDB showed the least significant reductions of total mean values for water regime. That response can be considered an acclimation mechanism to soil water deficit. Since plants can use physiological mechanisms for translocation and/or storage of assimilates in their organs during adverse periods, as shown by Catongo x SCA-6 (+14% SDB), SCA-6 x IMC-67 (+15% H, +22% CD and +44% SDB), MOC-01 x Catongo (+26% SDB) and RB-40 x MOC-01 (+12% CD and +43% SDB), which mean values were above the general mean for the trait. In general, water stress limits plant growth and developments. Studies with *Eucalyptus* and *Populus* indicated that drought affected the total vegetative growth [50, 51, 52], with height decreases of up to 25% in *Citrus* plants [64]. Thus, further studies are needed to obtain more information on the seasonal dynamics of *T*. *cacao* growth. Plants of this species exhibit alternation in shoot and root system growth phases [65].

It can be observed for the evaluated *T*. *cacao* progenies, that the R/S ratio and RD < 1 mm had mean increases of 7% and 18%, respectively, under conditions of soil water limitation. Recent studies demonstrated that drought tolerant *T*. *cacao* genotypes maintained a root growth similar to the control plants, showing greater amount of fine roots [39]. Other studies have indicated that under field conditions *T*. *cacao* plants produce also a large number of fine roots (RD < 1 mm), and their growth is directly related with rainfall frequency [65]. Under water stress conditions, metabolites are preferably partitioned to primary root elongation in order to increase water uptake [50, 52]. Such condition can increase photosynthate allocation to roots and, consequently, maintenance of the cellular water status for a longer period [66, 67, 68]. However, the spread of the root system (depth and lateral distribution) also depends on the physico-chemical characteristics of the soil, as well as available water content [69].

The observed differences between the means of *T*. *cacao* progenies for most morphological attributes analyzed indicate a very favorable condition for breeding. The heterogeneity of the genetic material allows selection, which in turn, may result in genetic gains from the identification of superior genotypes. Additionally, the high heritability (h^2^) observed for LDB (83%), LN (80%) TDB (80%), SDB (78%), RDB (77%), ILA (77%), RA (76%), SLA (73%), CD (70%), RV (69%) and RL (65%) suggests that these characters have potential to assist selection of *T*. *cacao* genotypes for tolerance to soil water deficit under greenhouse conditions. These results need to be validated under field conditions (Table 3).

Superiority of additive genetic effects (CGA) was found over non-additive genetic effects (SCA) in almost every morphological attribute analyzed. This suggests that the use of these parents in intrapopulational breeding programs can be an important strategy for improving expression of these characteristics. The superiority of the effects of GCA in terms of mean squares was also observed in other combining ability studies in *T*. *cacao* [70]. However, the significance for both GCA and SCA demonstrates the existence of variability due to both effects.

Significant effects (p < 0.05) between both GCA and SCA x water regimes, for most characters (Table 5), indicate that the assessment of stressed and control progenies favor identification of variability among genotypes. This variability may be related to additive (g_i_) and/or non-additive (s_ij_) genetic effects. However, the significant interaction progenies x water regime reduces the relationship between phenotype and its genotype, restricting the validity of inferences about the behavior, from the viewpoint of breeding and inheritance of quantitative traits [71]. In work with *Eucalyptus* clones submitted to different water regimes, significant differences were found among both clones and water regimes for most morphological and physiological traits [72].

For the growth and morphological variables RA, ILA, RD, RL, SLA and SDB, the effects of GCA x A was higher than the variance component of the interaction SCA x A, suggesting differential response among parents and little variation among hybrid combinations. Therefore, it is necessary to select different parents to cross for specific water regimes (Table 5). In contrast, for the variables H, CD, LDB, TDB, LAR, RDB, R/S, RD < 1 mm, 1 < RD < 2 mm and RD > 2 mm were only observed significant (p < 0.05) effects in the interaction SCA x water regimes, which allows to infer that there was differential response of hybrid combinations against water regimes and little variation between parents.

The parents SCA-6, IMC-67 and MOC-01 were those with the largest positive GCA values for the development of the root system, regardless of the water condition. In contrast, the PUCALA parent showed the highest negative values of GCA. The poor performance of PUCALA may be associated with genetic factors, since its progenies showed leaves with smaller dimensions than the others, resulting often in a smaller TLA, LN and LDB. A low estimate of the effects of GCA indicates that the value of the parent’s GCA, obtained based on its hybrid combinations and other parents do not differ much from the overall mean of the diallel population [46]. On the other hand, the higher these estimates, positive or negative, show evidence that the parent in question is far superior or inferior to the other parents of the diallel and can contribute (positive) for the increased expression of the character or reduction (negative value) of its manifestation.

The effect of the specific combining ability is interpreted as the deviation of a cross compared to what would be expected based on the GCA of their parents [46]. When the values are positive and negative, there is evidence of bidirectional dominance. Therefore, there are genes that enhance the expression of the character and others, equally dominant, that reduce it. Thus, the high SCA values (negative) for the progenies MOC-01 x IMC-67, RB-40 x IMC-67, PUCALA x IMC-67, MOC-01 x SCA-6, RB-40 x SCA-6 and Catongo x IMC-67 for most analyzed traits, suggest that these hybrid combinations may exhibit favorable factors that enable continued plant growth and development, even in adverse conditions of water availability.

The shoot growth attributes (LDB, CD and TLA) were not directly associated with root development (RL, RD < 1 mm, 1 < RD < 2 mm and RV) under stress conditions (Fig 2). This may be associated with reduction in water consumption or with mobilization of photosynthates for root development [67]. In contrast, in the control plants of the several *T*. *cacao* progenies the response was reverse, suggesting that under normal water availability conditions a functional balance between water uptake by roots and photosynthesis by shoots may occur. This functional balance can be altered if the water supply decreases [61].

## Conclusions

The SCA 6 genetic material showed high general combining ability for growth variables regardless of the water regime, meaning that the crosses in which participated, tend to provide greater accumulation of genes with favorable additive effect and can be considered in future parental interesting combinations. The growth variables CD, TLA, LDB, SDB, RDB, TDB, RL, RV, RD < 1 mm and 1 < RD < 2 mm were the variable that most contributed in the separation of *T*. *cacao* genotypes tolerant to water stress and can be used in selecting plants tolerant to drought.

## References

[1] Lambers H, Porter H (1992) Inherent variation in growth rate between higher plants: a search for physiological causes and ecological consequences. Advances in Ecological Research 23, 187–261.

[2] Chapin FS III, Autunm K, Pugnaire F (1993) Evolution of suites of traits in response to environmental stress. American Naturalist 142, 79–92.

[3] Almeida A-AF, Valle RR (2007) Ecophysiology of cacao tree. Brazilian Journal of Plant Physiology, 19, 425–448.

[4] Abo-Hamed S, Collin HA, Hardwick K (1983) Biochemical and physiological aspects of leaf development in cocoa (*Theobroma cacao* L.). Growth, orientation, surface structure and water loss from developing flush leaves. New Phytologist 95, 7–9.

[5] Balasimha D, Daniel EV, Prakash B (1991) Influence of environmental factor on photosynthesis in cacao tree. Agricultural and Forest Meteorology 55, 15–21.

[6] Belsky JM, Siebert SF (2003) Cultivating Cacao: Implications of Sun-Grown Cacao on Local Food Security and Environmental Sustainability, 20, 277–285.

[7] Bae H, Kim SH, Kim MS, Sicher RC, Lary D, Strem MD, et al (2008) The drought response of *Theobroma cacao* L (cacao) and the regulation of genes involved in polyamine biosynthesis by drought and other stresses. Plant Physiology and Biochemistry, 46, 74–188. 10.1016/j.plaphy.2007.10.014 18042394

[8] Gramacho ICP, Magno AES, Mandarino EP, Matos A (1992) Cultivo e beneficiamento do cacau na Bahia. Ilhéus: Ceplac, 124 p.

[9] Anim-Kwapong G, Frimpong E (2005) Vulnerability of agriculture to climate change impact of climate change on cocoa production. Accra, Ghana.

[10] Almeida A-AF, Brito RCT, Aguilar MAG, Valle RR (2002) Water relations aspects of *Theobroma cacao* L. clones. Agrotrópica, 14, 35–44.

[11] Balasimha DV, Rajagopal (1988) Stomatal responses of cocoa (*Theobroma cacao* L) to climatic factors. Indian Journal of Agricultural Sciences, 58, 213–216.

[12] Rada F, Jaimez RE, Garcia-Nuñez C, Azocar A, Ramírez M (2005) Water relations in *Theobroma cacao* var. Guasare under periods of water deficits. Revista de la Facultad de Agronomia de la Universidad del Zulia 22, 112–120.

[13] Carr MKV, Lockwood G (2011) The water relations and irrigations requirements of cocoa (*Theobroma cocoa* L.): A Review, Experimental. Agriculture, 47, 653–676.

[14] Padi FK, Adu-Gyamfi P, Akpertey A, Alfred A, Ofori A (2013) Differential response of cocoa (*Theobroma cacao* L.) families to field establishment stress. Plant Breeding 132, 229–236.

[15] Moser G, Leuschner Ch, Hertel D, Hölscher D, Köhler M, Leitner D, et al (2010) The response of cacao trees (*Theobroma cacao* L) to a 13-month desiccation period in Sulawesi, Indonesia. Agroforestry Systems 79, 171–187.

[16] Sale PJM (1970) Growth, flowering and fruiting of cacao under controlled soil moisture conditions. Journal of Horticultural Science 45, 99–118.

[17] Mohd Razi I, Mohd Kamil Y, Marziah M (1992) Growth, water relation, stomatal conductance and proline concentration in water stressed banana. Asian Journal of Plant Sciences 6, 709–713.

[18] Gao HJ, Yang HQ, Wang JX (2009) Arginine metabolism in roots and leaves of apple (*Malus domestica* Borkh.) The tissue-specific formation of both nitric oxide and polyamines. Scientia Horticulturae 119, 147–152.

[19] Maurel C, Simonneau T, Sutka M (2010) The significance of roots as hydraulic rheostats. Journal of Experimental Botany, 61, 3191–3198. 10.1093/jxb/erq150 20522526

[20] Davies WJ, Bacon MA (2003) Adaptation of root to drought In: Root Ecology. (De Kroon H. and Visser E. J.W., Eds.). Springer-Verlag, Berlin, Germany 173–192.

[21] Fageria N. K. (2012). The Role of Plant Roots in Crop Production, CRC Press Boca Raton FL.

[22] Zuidema PA, Leffelaar PA, Gerritsma W, Mommer L, Anten NPR (2005) A physiological production model for cocoa (*Theobroma cacao* L): model presentation, validation and application. Agricultural Systems, 84, 195–225.

[23] Orchard JE, Saltos MR (1988) The growth and water status of cocoa during its first year of establishment under different methods of soil water management. In: Proceedings of 10th international cocoa research conference, Santo Domingo, Dominican Republic, 193–198.

[24] Moroni MT, Worledge D, Beadle CL (2003) Root distribution of *Eucalyptus nitens* and *Eucalyptus globulus* in irrigated and droughted soil. Forest Ecology and Management, Amsterdam, 177 (1), 399–407.

[25] Yin C, Peng Y, Zang R, Zhu Y, Li C (2005) Adaptive responses of *Populus kangdigensis* to drought stress. Physiologia Plantarum, 123, 445–451.

[26] Damatta FM, Chaves ARM, Pinheiro HA, Ducatti C, Loureiro ME (2003) Drought tolerance of two field-grown clones of *Coffea canephora*. Plant Science, 164, 111–117.

[27] Muller B, Pantin F, Génard M, Turc O, Freixes S, Piques M, et al (2011) Water deficits uncouple growth from photosynthesis, increase C content, and modify the relationships between C and growth in sink organs. Journal of Experimental Botany, 62, 1715–1729. 10.1093/jxb/erq438 21239376

[28] Figueiroa JM, Barbosa DCA, Simabukuro EA (2004) Crescimento de plantas jovens de *Miracrodruon urundeuva* Allemão (Anacardiaceae) sob diferentes regimes hídricos. Acta Botanica Brasilica, 18, 573–580.

[29] Bartley BGD (2005) The Genetic diversity of cacao and its utilization. Wallingford: CABI Publishing

[30] Cruz CD, Regazzi AJ (2004). Modelos biométricos aplicados ao melhoramento genético. Viçosa: UFV 480 p

[31] Jaramillo G, Morante N, Pérez JC, Calle F, Ceballos H, Arias B, et al (2005) Diallel analysis in cassava adapted to the middle-altitude valleys environments. Crop Science, 45, 1058–1063.

[32] Efombagn MIB, Sounigo O, Nyassé S, Manzanares-Dauleux M, Eskes AB (2009) Phenotypic variation of cacao (*Theobroma cacao* L.) on farms and in the gene bank in Cameroon. Journal of Plant Breeding and Crop Science, 1, 258–264.

[33] Efombagn MIB, Motamayor JC, Sounigo O, Eskes AB, Nyassé S, Cilas C, et al (2008) Genetic diversity and structure of farm and gene bank accessions of cacao (*Theobroma cacao* L.) in Cameroon revealed by microsatellite markers. Tree Genetics and Genome 4, 821–831.

[34] Efombagn MIB, Sounigo O, Nyassé S, Manzanares-Dauleux M, Cilas C, Eskes AB, et al (2006) Genetic Diversity in cocoa germplasm of southern Cameroon revealed by simple sequences repeat (SSRs) markers. African Journal Biotechnol 5, 1441–1449.

[35] Dias LAS, Marita J, Cruz CD, Barros EG, Salomão TMF (2003) Genetic distance and its association with heterosis in cacao. Brazilian Archives of Biology and Technology, 46, 339–347.

[36] Dias LAS, Kageyama PY (1997) Multivariate genetic divergence and hybrid performance of cacao (*Theobroma cacao L*.*)*. Brazilian Journal of Genetics, Ribeirão Preto 20, 63–70.

[37] Dias LAS, Kageyama PY (1997) Temporal stability of multivariate genetic divergence in cacao (*Theobroma cacao* L.) in Southern Bahia conditions. Euphytica 93, 181–187

[38] Dias LAS, Kageyama PY, Castro GCT (1997) Divergência fenética multivariada na preservação de germplasma de cacau (*Theobroma cacao* L.). Agrotrópica, 9, 29–40.

[39] Santos IC, Almeida AAF, Anhert D, Conceição AS, Pirovani CP, Pires JL, et al (2014) Molecular, Physiological and Biochemical Responses of *Theobroma cacao* L. Genotypes to Soil Water Deficit. PlosOne, 9: e115746. 10.1371/journal.pone.0115746 PMC4277404 25541723

[40] Arévalo AR, Soria J (1975) Evaluación de cuatro métodos de polinización artificial en el aumento de producción de cacao (Theobroma cacao L.). In: Proceedings of the 5th International Cocoa Research Conference. Ibadan, Nigeria, 78–94.

[41] Souza JO Junior, Carmello QAC (2008) Formas de adubação e doses de uréia para mudas clonais de cacau cultivadas em substrato. Revista Brasileira Ciência do Solo 32, 2367–2374.

[42] Scholander PF, Hammel HT, Bradstreet ED, Hemmingsen EA (1965) Sap pressure in vascular plants.Science, 148, 339–46. 17832103 10.1126/science.148.3668.339

[43] Kummerow J, Kummerow M, Silva WS (1982). Fine-root growth dynamics in cacao (*Theobroma cacao* L). Plant Soil 65, 193–201.

[44] Hunt R (1990) Basic Growth analysis: Plant growth analysis for beginners. Unwin Hyman London.

[45] Lynch M, Walsh B (1998) Genetics and Analysis of Quantitative Traits. Sinauer Associates, Sunderland, MA.

[46] Griffing B (1956) Concept of general and specific combining ability in relation to diallel crossing system. Australian Journal of Biology 9, 463–493.

[47] Software R (2014) The R Foundation for Statistical Computing. http://www.r-project.org.

[48] Hair JF Jr, Black WC, Babin BJ, Anderson RE (2010). Multivariate data analysis (7th ed). Upper Saddle River, NJ: Prentice Hall.

[49] Yan W, Rajcan I (2002) Biplot analysis of teste sites and trait relations of soybean in Ontario. Crop Science, 42, 11–20. 11756248 10.2135/cropsci2002.1100

[50] Li C, Wang K (2003) Differences in drought responses of three contrasting *Eucalyptus microtheca* F. Muell. populations. Forest Ecology and Management 179, 377–385.

[51] Osório J, Osório ML, Chaves MM, Pereira JS (1998) Water deficits are more important in delaying growth than in changing patterns of carbon allocation in *Eucalyptus globulus*. Tree Physiology 18, 363–373. 12651361 10.1093/treephys/18.6.363

[52] Yin C, Wang X, Duan B, Luo J, Li C (2005) Early growth dry matter allocation and water use efficiency of two sympatric *Populus* species as affected by water stress. Environmental and Experimental Botany 53, 315–22.

[53] DaMatta FM (2004) Exploring drought tolerance in *coffee*: a physiological approach with some insights for plant breeding. Brazilian Journal of Plant Physiology, 16, 1–6.

[54] Damatta FM, Chaves ARM, Pinheiro HA, Ducatti C, Loureiro ME (2003) Drought tolerance of two field-grown clones of *Coffea canephora*. Plant Science, 164, 111–117.

[55] Joly R, Hahn D (1989) An empirical model for leaf expansion in cacao in relation to plant water deficit. Annals of Botany, 64, 1–8.

[56] Frimpong E, Adu-Ampomah Y, Karimu A (1996) Efforts to breed for drought resistant cacao in Ghana. Proceedings of the 12th International Cacao Research Conference, Bahía, Brasil, 24–25.

[57] Hadley P, Pearson S (1996) A physiologist’s view of cacao yield. Proceedings of the Malaysian International Cacao Conference, Kuala, Lumpur, Malaysia 194–209.

[58] Merchant A, Callister A, Arndt S, Tausz M, Adams M (2007) Contrasting physiological response of six *Eucalyptus* species to water deficit. Annals of Botany,100, 1507–1515 17905722 10.1093/aob/mcm234 PMC2759221

[59] Albacete AA, Martínez-Andújar C, Pérez-Alfocea F, (2014) Hormonal and metabolic regulation of source-sink relations under salinity and drought: From plant survival to crop yield stability. Biotechnology Advances, 32, 12–30. 10.1016/j.biotechadv.2013.10.005 24513173

[60] Kozlowski TI (1976) Water supply and leaf shedding In: Water stress and plant growth. New York; Academic Press 4, 191–222.

[61] Ballester C, Castel J, Intrigliolo DS, Castel JR (2011) Response of *Cementinade nules* citrus trees to summer deficit irrigation. Yield components and fruit composition. Agricutltural Water Management, 98, 1027–1032.

[62] Seyed YS, Lisar RM, Mosharraf M, Hossain MMR, Ismail MMR (2012) Water Stress in Plants: Causes, Effects and Responses, 300.

[63] Silva W, Sediyama T, Silva AA, Souza AP (2001) Taxa fotossintética líquida de *Eucalyptus citriodora* Hook *e E*. *grandis* W.Hill em níveis de água no solo e associação com *Brachiaria brizantha* Staf. Acta Scientiarum, Maringá, 23, 1205–1209.

[64] Ortuño MF, Alarcón JJ, Nicolás E, Torrecilla A (2004) Interpreting trunk diameter changes in young lemon trees under deficit irrigation. Plant Science, 167, 275–280.

[65] Silva W, Kummerow J (1998) Fine-root growth and longevity in a cacao (*Theobroma cacao* L.) plantation. Agrotrópica, 10, 31–34.

[66] Skirycz A, Inze D (2010) More from less: plant growth under limited water. Curr Opin Biotechnol 21, 197–203. 10.1016/j.copbio.2010.03.002 20363612

[67] Moroni MT, Worledge D, Beadle CL (2003) Root distribution of *Eucalyptus nitens* and *Eucalyptus globulus* in irrigated and droughted soil. Forest Ecology and Management, Amsterdam, 177 (1), 399–407.

[68] Blum A (2005) Drought resistance, water-use efficiency, and yield potential: are they compatible, dissonant, or mutually exclusive? Australian Journal of Agricultural Research 56, 1159–1168.

[69] Muller B, Pantin F, Génard M, Turc O, Freixes S, Piques M, et al (2011) Water deficits uncouple growth from photosynthesis, increase C content, and modify the relationships between C and growth in sink organs. J Exp Bot. 62, 1715–1729. 10.1093/jxb/erq438 21239376

[70] Dias LAS, Kageyama PY (1995) Combining-ability for cacao (*Theobroma cacao* L.) yield components under southern Bahia conditions. Theoretical and Applied Genetics, 90, 534–541. 10.1007/BF00222000 24173948

[71] Li C, Berninger F, Koskela J, Sonninen E (2000) Drought responses of *Eucalyptus microtheca* provenances depend on seasonality of rainfall in their place of origin. Australian Journal of Plant Physiology, 27, 231–238.

[72] Vellini ALTT, Paula NF, Alves PLC, Pavani C, Bonine CAV, Scarpinati EA, et al (2008) Respostas fisiológicas de diferentes clones de eucalipto sob diferentes regimes hídricos. Revista Árvore, Viçosa—MG,32, 651–663.

